# Evaluation of the Interaction between Phosphohistidine Analogues and Phosphotyrosine Binding Domains

**DOI:** 10.1002/cbic.201402090

**Published:** 2014-04-25

**Authors:** Tom E McAllister, Katherine A Horner, Michael E Webb

**Affiliations:** [a] School of Chemistry and Astbury Centre for Structural Molecular Biology, University of Leeds Woodhouse Lane, Leeds, LS2 9JT (UK) E-mail: m.e.webb@leeds.ac.uk Homepage: http://www.chem.leeds.ac.uk/MEW/

**Keywords:** cell signaling, phosphohistidine, phosphotyrosine, protein modifications, synthetic analogues

## Abstract

We have investigated the interaction of peptides containing phosphohistidine analogues and their homologues with the prototypical phosphotyrosine binding SH2 domain from the eukaryotic cell signalling protein Grb2 by using a combination of isothermal titration calorimetry and a fluorescence anisotropy competition assay. These investigations demonstrated that the triazole class of phosphohistidine analogues are capable of binding too, suggesting that phosphohistidine could potentially be detected by this class of proteins in vivo.

Phosphorylation of proteins is well established as a dominant mechanism of signal transduction in eukaryotic cells.[1] Phosphorylation occurs in response to extracellular and intracellular signals, and the modified protein can then be sensed by sequence-specific modular binding domains.[2] These include SH2 and PTB domains, which bind phosphotyrosine,[3] and 14-3-3 and WW domains, which bind phosphothreonine and phosphoserine.[4] In contrast, it is still unknown how, or indeed whether, other phosphorylated amino acids such as phosphohistidine are sensed. The lability of this latter modification means that it has only been directly observed in a limited subset of eukaryotic proteins, including the cystic fibrosis transmembrane receptor (CFTR),[5] histone H4[6] and the potassium-activated calcium channel KCa3.1.[7] In the first and last cases, histidine phosphorylation directly activates the channel; however, in the case of histone H4 and many others, the function has not been determined. Although proteomic methods[8] are increasingly able to identify this modification in proteins, no general function for it has been identified.

We recently reported the synthesis of a triazolyl analogue, **3 a**, of τ-phosphohistidine (**1**, Scheme 1), which is fully compatible with the Fmoc-protection strategy for solid-phase synthesis.[9] Independently, Kee et al. have demonstrated that, following Boc-peptide synthesis, it was possible to use analogue **2** as a hapten to identify antibodies capable of identifying phosphohistidine in a site-specific fashion.[10] More recently a truncated form of **2** has been used to generate a polyclonal antibody that is able to identify **1** in a variety of proteins.[11]

**Scheme 1:**
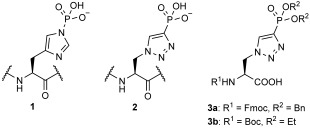
Structures of τ-phosphohistidine (1) and the corresponding triazole-phosphonate mimic, 2, which can be incorporated into peptides by using precursors 3 a and 3 b.

The study of phosphoprotein interactions in vitro requires a source of phosphorylated protein. There are well known techniques for isolating phosphate ester-containing proteins, but maintaining phosphorylated histidine residues during protein isolation is notoriously difficult,[12] even though histidine can be chemically phosphorylated in vitro.[13] An alternative approach is the incorporation of phosphoamino acids (or precursors) into recombinant proteins. This approach was used by Serwa et al. to generate an analogue of phosphotyrosine **4** by Staudinger phosphite reaction on a genetically incorporated *p*-azidophenylalanine to form phosphoramidate **5** (Scheme 2).[14] A further phosphotyrosine analogue, *p*-carboxymethylphenylalanine (**6**),[15] has also been incorporated by an amber-suppression approach; direct genetic incorporation of phosphoserine has also been achieved.[16]

**Scheme 2:**
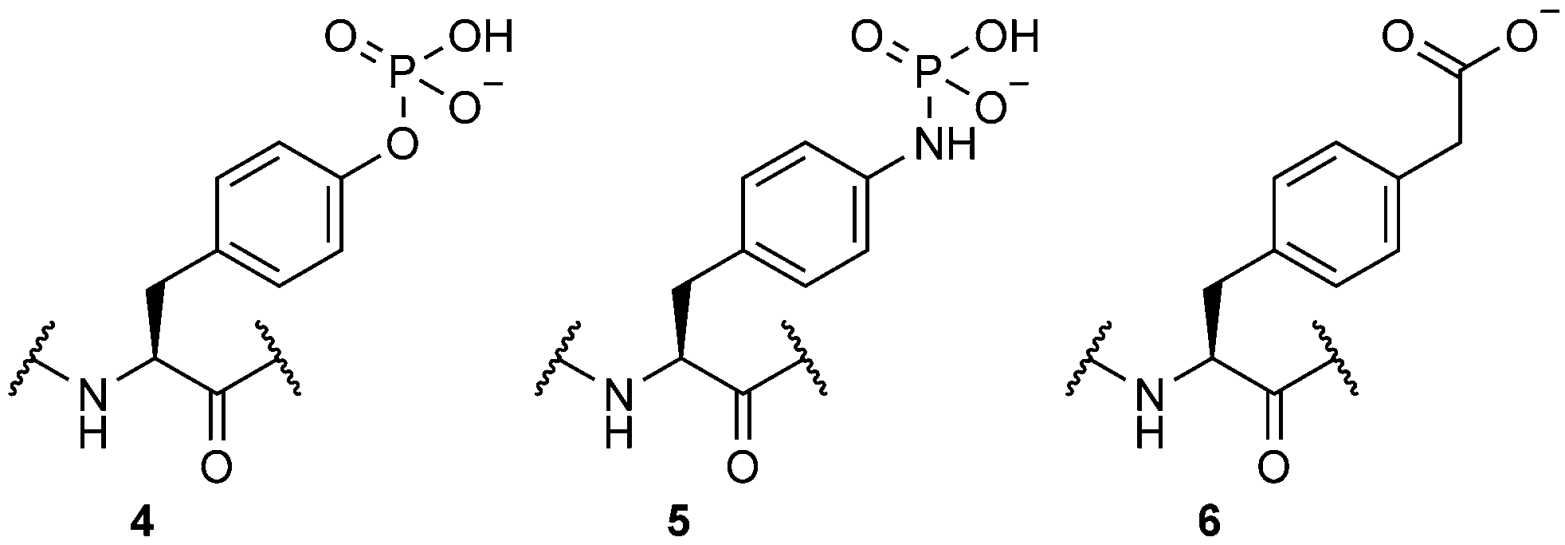
Structures of phosphotyrosine 4 and analogues 5 and 6, which have previously been incorporated into recombinant proteins.

We hypothesised that we could generate a protein containing our phosphohistidine analogue by first producing a protein with an azidoalanine residue and subsequent reaction with a suitably protected alkynyl phosphonate **8**. Unfortunately l-azidoalanine cannot be incorporated by amber suppression and is only poorly incorporated as a methionine surrogate.[17] However, the homologous compound, l-azidohomoalanine (**7**), is well accepted as a methionine surrogate[18] and reaction of this residue with alkynyl phosphonate **8** would ultimately produce homotriazole **10** (Scheme 3). Though this triazole could perhaps no longer mimic phosphohistidine, it occurred to us that **10** could present the phosphoryl group in a similar orientation to phosphotyrosine (due to the extra flexibility bestowed by the γ-methylene group) and thus constitute a novel analogue of phosphotyrosine **4**. Though not our primary goal, investigation of this seemed prudent as, if a suitable mimic, **10** would be a more accessible phosphotyrosine analogue than the current alternatives.

**Scheme 3:**
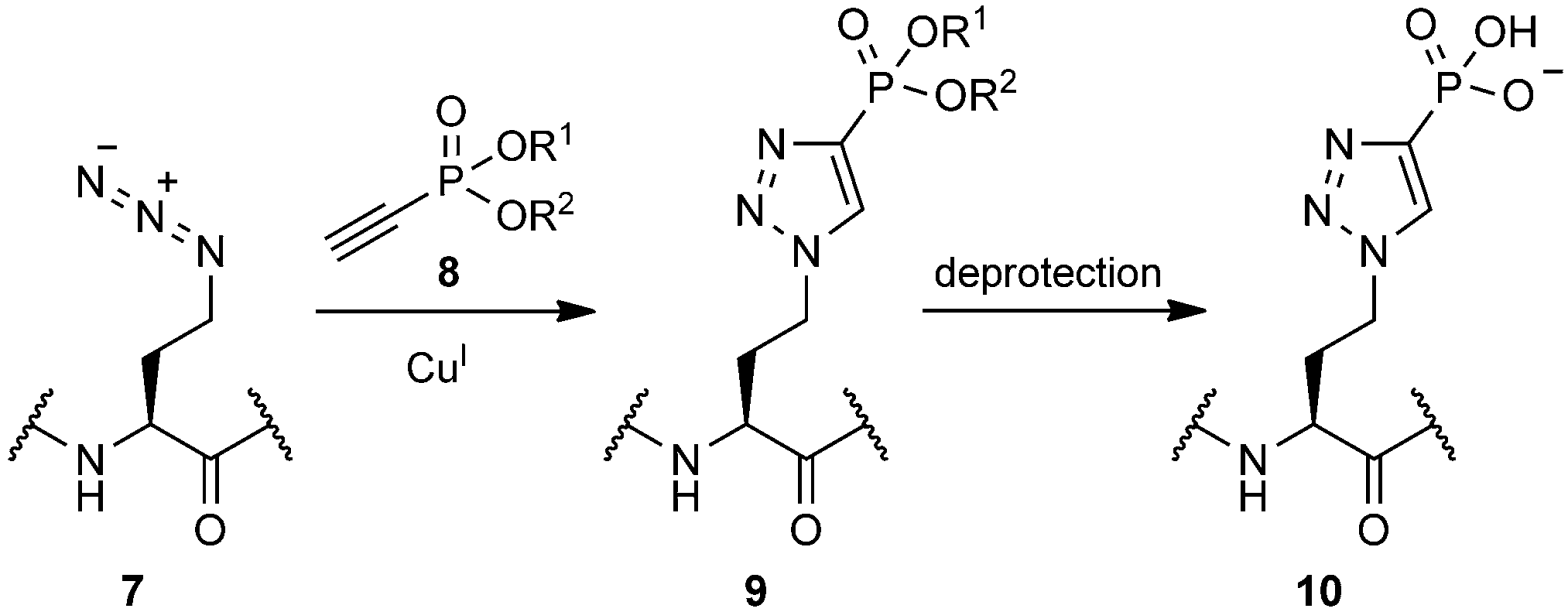
Proposed route to generate a protein containing the putative phosphotyrosine analogue 10.

We envisaged producing suitable peptides by Fmoc-SPPS and investigating the interaction with proteins known to bind to phosphotyrosine. Our initial test system was the prototypical SH2 phosphotyrosine binding domain from Grb2.[19] This domain has been well characterised, binding with a high affinity (*K*_d_=200 nm) to the Shc-derived phosphotyrosine-containing hexapeptide **11** (Scheme 4).[20] We generated the required Fmoc-/benzyl-protected amino acid **14** from Fmoc-protected homoazidoalanine **12** and dibenzyl phosphonylacetylene **13** through a [3+2] cycloaddition in an analogous manner to our previously reported synthesis of Fmoc-/benzyl-protected **3 a** (Scheme 4).[9] Fmoc-SPPS was then used to generate peptides **11** and **15**, which contain phosphotyrosine (pY) **4** and homotriazole (phTz) **10**, respectively. We also investigated using an Fmoc-/ethyl-protected derivative of **14** but encountered deprotection difficulties (as reported previously[21]), so this approach was not pursued further.

**Scheme 4:**
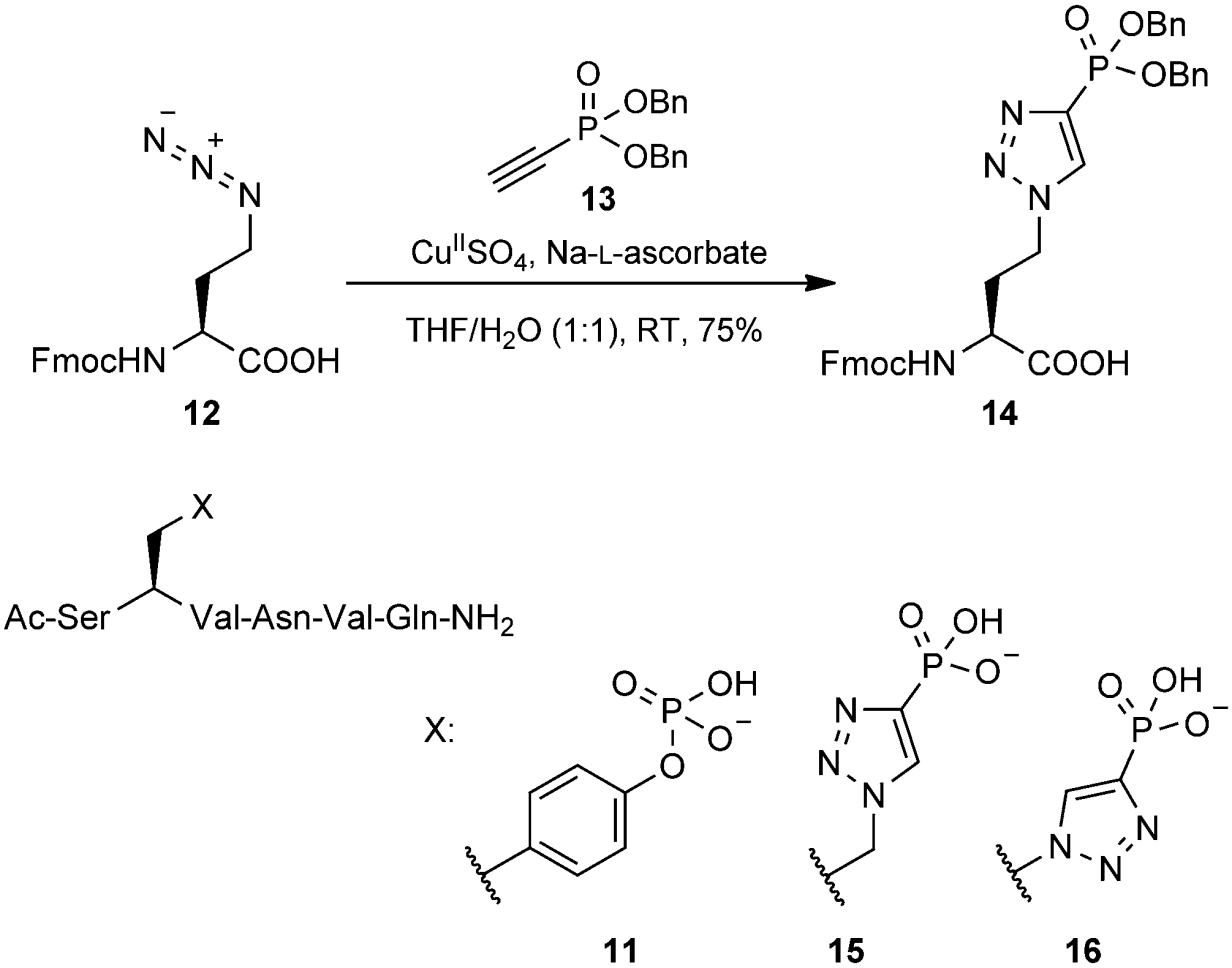
Synthesis of Fmoc-/benzyl-protected homotriazole 14 from Fmoc-azidohomoalanine 12 and alkyne 13, as well as peptides 11, 15 and 16, which were used in binding experiments. For full experimental procedures see the Supporting information.

For our initial assays of ligand–protein interactions, we used full-length Grb2 that was purified as a glutathione S-transferase (GST) fusion. Titration of the pY control peptide **11** into the protein yielded a sigmoidal binding curve (see the Supporting Information) corresponding to an affinity of (196±21) nm, consistent with literature reports. The titration of phTz peptide **15** into the protein was inconclusive; although the interaction was clearly weak it was not possible to rule out an interaction. Protein aggregation prevented experiments at higher concentrations, and attempts to cleave the GST-tag enzymatically were unsuccessful. To overcome this hurdle, the SH2 domain was subcloned into pET28a to obtain an N-terminally His-tagged isolated SH2 domain. The protein overexpressed well in the insoluble fraction, which was solubilised in 8 m urea and purified by immobilised metal-affinity chromatography. On-column refolding then yielded pure soluble protein, but size-exclusion analysis of this protein revealed two distinct non-interconverting species with identical appearance when analysed by SDS-PAGE (Figure S4 in the Supporting Information). ITC assay of peptide **11** binding to the two protein fractions revealed that the larger (earlier-eluting) species had a significantly reduced affinity (4.8±1.2) μm versus (344±42) nm for the smaller species (Figure S8). These data suggested that the early peak represents a domain-swapped dimer (as previously observed by Benfield et al.)[22] and so the later peak, corresponding to monomeric His_6_-(Grb2-SH2) was used in all subsequent assays, this allowed experiments to be conducted at significantly higher (more than tenfold) protein concentrations.

The interaction of phTz peptide **15** with Grb2-SH2 was analysed by ITC. Initial experiments under our standard conditions (final ratio of ligand to protein 2:1) were inconclusive, thus suggesting a possible poorly defined weak binding (not shown). The experiment was therefore repeated under low *c*-value conditions[23] with a final ratio of ligand to protein of 8:1 (Figure 1 B). This titration was indistinguishable from a titration of the peptide into buffer alone, conclusively confirming no binding. This was somewhat disappointing and surprising given that recent work by Hofmann et al. revealed that the Src kinase-SH2 domain can bind to phosphoarginine.[24] However, this led us to consider that the Grb2-SH2 domain might bind to other proteinogenic phosphoamino acids. To determine if τ-phosphohistidine **1** would bind, we synthesised a further peptide, **16**, that contained our phosphotriazole (pTz) analogue **2**. Again, an initial ITC experiment under standard conditions was inconclusive (data not shown), but titration to a final ligand to protein ratio of 8:1 (Figure 1 C) yielded a clear binding interaction. This was readily distinguishable from the buffer dilution and corresponded to 1:1 binding with *K*_d_=(719±28) μm (Figure 1). It has been shown that a 20-fold change in affinity can be attributed to a change in protonation state.[25] The relatively low affinity of the peptide **16**:Grb2-SH2 interaction observed in our study might be due to a difference in protonation states between the phosphate of **4** and the phosphonate of **2**. To investigate this, we conducted NMR titrations (see the Supporting Information) that revealed the p*K*_a_ of **4** to be 5.8 and **2** to be 5.95. Hence, under the conditions of our experiments (pH 7.4) both phosphoryl groups should be di-anionic, so it is unlikely that the difference in affinity is due to differing protonation states.

**Figure 1:**
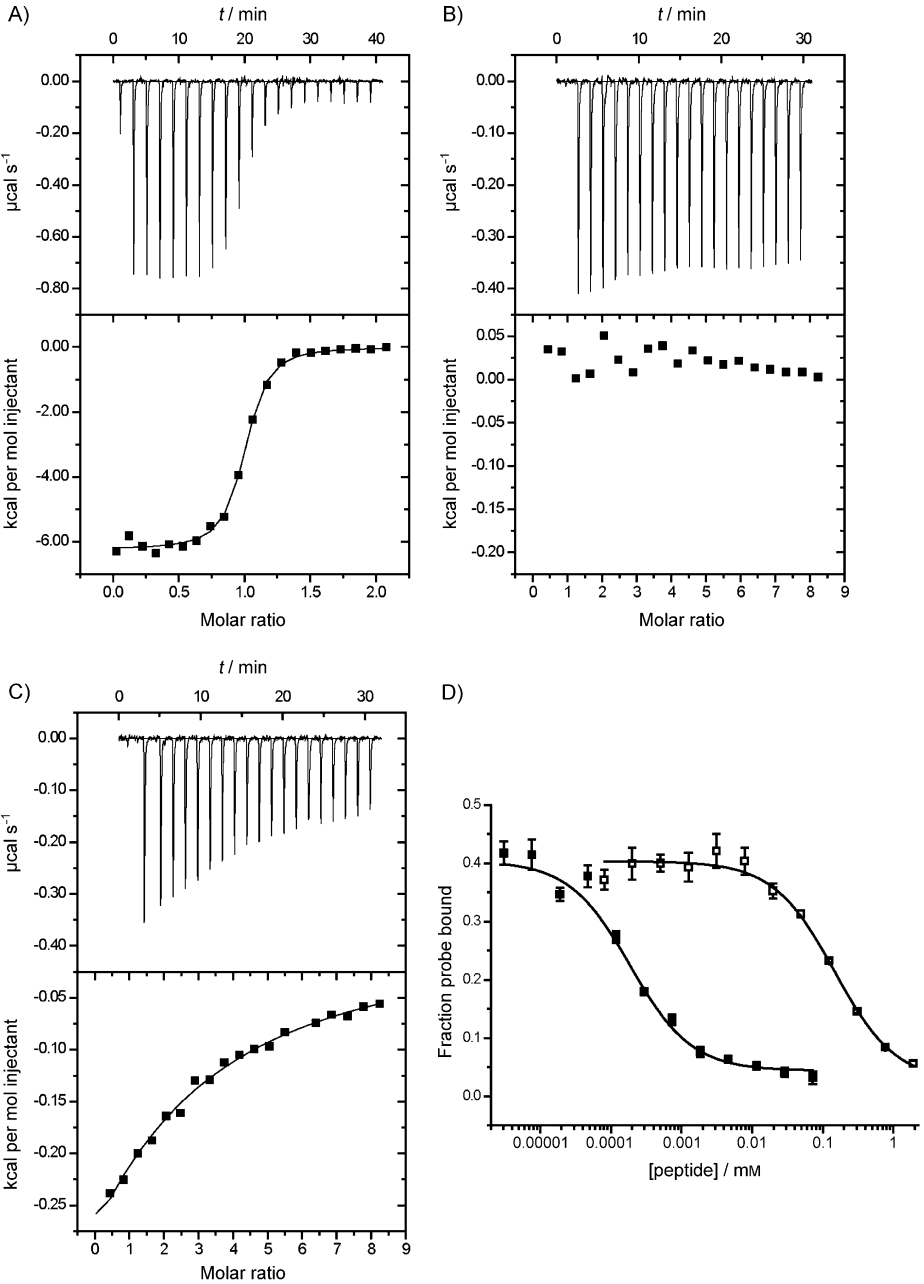
Isothermal titration calorimetry of peptides A) 11, B) 15 and C) 16 into the Grb2 SH2 domain reveals equilibrium binding constants of (385±41) nm for 11 and (719±28) μm for 16. D) A competitive fluorescence polarisation assay indicates an IC_50_ of (442±80) nm for pTyr peptide 11 (▪) and (363±15) μm for pTz peptide 16 (□). See the Supporting Information for control dilution experiments and full fitting of polarisation data.

To investigate the binding of other phosphoamino acids and confirm that peptides **11** and **16** were binding at the same site, we developed a competition fluorescence anisotropy assay. Titration of a phosphotyrosine-containing peptide with an N-terminal fluorescein, **17**, with purified His_6_-(Grb2-SH2) (see Figure S11) yielded a binding constant of (283±18) nm; this is consistent with the *K*_d_ previously measured by ITC. We then conducted competition experiments on all three peptides used for ITC together with peptides **18**–**21** (AcHN-Ser-Xaa-Val-Asn-Val-Gln-NH_2_ Xaa=Tyr, His, pSer or pThr, see the Supporting Information). Binding (displacement of **17**) was observed only for peptides **11** and **16**, which contain pTyr and pTz, respectively (Figure 1 D). A slightly lower than expected IC_50_ of 360 μm was observed for the pTz peptide; this suggests that the ITC analysis might have underestimated the affinity of this peptide. No evidence for binding was observed for the other peptides (Figure S11); this suggested that the Grb2-SH2 domain will not simply bind to a phosphoryl group presented in the correct peptide context, or to an aromatic ring.

We have shown that a proven τ-phosphohistidine mimic is capable of sequence-specific binding to a canonical phosphotyrosine-binding domain. Phosphotyrosine antibodies have previously been shown to bind to phosphohistidine,[8b] but our findings contradict previous studies of phosphohistidine-containing peptides binding to SH2 domains (however the conditions used in these studies precluded the identification of low-affinity binding such as we have observed and used a different SH2 domain, from phospholipase C-γ1).[26] Phosphotyrosine binding protein modules are a common feature of human signalling proteins and, in many cases, the sequence specificity of these domains has not been fully elucidated. The affinities of known peptides for their target proteins also vary hugely, ranging from low nanomolar to high micromolar.[27] Low affinity therefore does not mean low significance; it is possible that a subset of assigned phosphotyrosine binding modules could additionally/alternatively interact with phosphohistidine-containing proteins. Further study to establish the generality of this observation is on-going.

## Experimental Section

Experimental details are given in the Supporting Information.

## Supporting Information

As a service to our authors and readers, this journal provides supporting information supplied by the authors. Such materials are peer reviewed and may be re-organized for online delivery, but are not copy-edited or typeset. Technical support issues arising from supporting information (other than missing files) should be addressed to the authors.

miscellaneous_information
