# Supplementary material for: Evaluation of the Interaction between Phosphohistidine Analogues and Phosphotyrosine Binding Domains
Source: Chembiochem. 2014 Apr 25;15(8):1088–91. doi: 10.1002/cbic.201402090 (PMC4159583; doi:10.1002/cbic.201402090)
Supplement: Supplementary file 1 — miscellaneous_information [file cbic0015-1088-sd1.pdf]

CHEMBIOCHEM

Supporting Information

© Copyright Wiley-VCH Verlag GmbH & Co. KGaA, 69451 Weinheim, 2014

Evaluation of the Interaction between Phosphohistidine Analogues and Phosphotyrosine Binding Domains

Tom E. McAllister, Katherine A. Horner, and Michael E. Webb*[a]

cbic_201402090_sm_miscellaneous_information.pdf

Supplementary Information

Page 2 Chemical synthesis

Page 6 Protein expression and purification

Page 10 Isothermal titration calorimetry

Page 13 pKa determination

Page 14 Fluorescence polarization methodology

Page 16 HPLC of peptides

Page 22 NMR spectra of selected compounds and peptides.

Chemical Synthesis

Fmoc-azidohomoalanine-OH **12** was purchased from Iris Biochem, other reagents were purchased from Sigma Aldrich, Alfa Aesar, Merck or Fisher Scientific and all were used without further purification. All solvents used were HPLC grade and mixtures are *v/v*. NMR data were collected using a Bruker DPX300 and analysed using MestReNova software. The following abbreviations are used to describe the multiplicity of signals: s = singlet, d = doublet, dd = doublet of doublets, qn = quintet, m = multiplet. J-values are given in Hz. IR spectra were recorded using a PerkinElmer spectrum one FTIR spectrometer. Optical rotations were measured using a Schmidt+Haensch polartronic H532, $[\alpha]_D$ values are given in 10^{-1} deg cm^2 g^{-1}. High resolution mass spectrometry (HRMS) was carried out on a Bruker Daltonics micrOTOF using electrospray ionisation. Column chromatography was carried out using silica gel and TLC was performed on silica gel 60-F_{254} (Merck) with detection by fluorescence upon irradiation with UV light or staining by immersion in a solution of potassium permanganate and heating.

Synthesis of Fmoc-phTz(OBn)$_2$-OH 14

(2*S*)-4-(4-dibenzylphosphonyl-[1,2,3]-triazol-1-yl)-2-(9H-fluoren-9-ylmethoxycarbonyl)-amino-butyrate

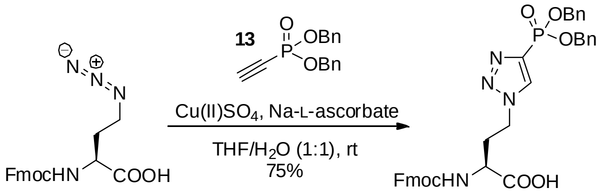

12 14

A freshly prepared solution of copper (II) sulfate (26 mg, 0.16 mmol) and sodium ascorbate (90 mg, 0.45 mmol) in H_2O (10 ml) was added to a stirred solution of dibenzyl ethynylphosphonate **13**[1] (430 mg, 1.50 mmol) and Fmoc-azidohomoalanine-OH **12** (366 mg, 1 mmol) in THF (10 ml) and the mixture stirred at rt for 70 min, at which time TLC showed complete consumption of the azide. The reaction mixture was diluted with 10% $Na_2CO_{3\ aq}$ (30 mL) and extracted with ether (2 × 20 mL). The aqueous phase was acidified to pH 1 by dropwise addition of conc. $HCl_{(aq)}$ and extracted with EtOAc (3 × 30 mL). The EtOAc extracts were combined, dried ($MgSO_4$) and concentrated *in vacuo* to yield a clear yellow oil. Column chromatography (DCM:MeOH:AcOH, 94:5:1) gave a colourless oil, which was dissolved in 1,4-dioxane and lyophilised to yield *Fmoc-phTz(OBn)$_2$-OH* **157** as an amorphous colourless solid (489 mg, 0.75 mmol, 75%). $[\alpha]_D^{22}$ + 22.5 (*c* 1.1, $CHCl_3$); δ_H (300 MHz, MeOD): 8.21 (1H, s, Trz-H_5), 7.66 (2H, d, $^3J_{H-H}$ 7.09, 2 × Fmoc-H_5), 7.54 (2H, d, $^3J_{H-H}$ 6.36, 2 × Fmoc-H_2), 7.32-7.10 (14H, m, 2 × Fmoc-H_4 + 2 × Fmoc-H_3 + 10 × Ph-H), 5.01 (4H, d, $^3J_{H-P}$ 8.78, 2 × OCH_2Ph), 4.49-4.38 (2H, m, 2 × CH_γ), 4.26 (2H, d, $^3J_{H-H}$ 6.46, Fmoc-CH_2), 4.09 (2H, d, $^3J_{H-H}$ 6.46, Fmoc-CH), 4.01 (1H, dd, $^3J_{H-H}$ 9.82, $^3J_{H-H}$ 4.32, CH_α), 2.75-2.32 (1H, m, CH_β), 2.20-2.03 (1H, m, C$H_{\beta'}$); δ_C (75 MHz, $CDCl_3$): 173.2 (s, *C*OOH), 156.3 (s, NH*C*OO), 143.8 (s), 143.6 (s), 141.3 (d, $^3J_{C-P}$ 1.9, Ph-C_1), 136.2 (d, $^1J_{C-P}$ 243.7, Trz-C_4), 135.4 (s), 135.3 (s), 131.8 (d, $^2J_{C-P}$ 34.3, Trz-C_5),128.6 (s), 128.1 (s), 127.8 (s), 127.1 (s), 125.1 (s, Fmoc-C_2), 120.0 (s, Fmoc-C_5), 68.9 (d, $^3J_{C-P}$ 5.6, OCH_2Ph) 67.0 (s, Fmoc-CH_2), 51.5 (s, C_α), 46.9 (s, Fmoc-*C*H), 47.1 (s, C_γ), 32.7 (s, C_β); δ_P (121 MHz, MeOD) 8.30 (qn,$^3J_{P-H}$ 8.78); ν_{max} (solid)/cm^{-1} 3313 (N-H stretch), 2958 (N-H stretch), 2586(O-H), 1729 (C=O), 1514 (N-H bend), 1211 (P=O), 1009 (P-O), 873, 740. *m/z*: Found *MNa*$^+$ 675.1969, $C_{35}H_{33}N_4NaO_7P$ requires 675.1979.

Peptide Synthesis

Resins and amino acids were purchased from Novabiochem: Fmoc-Asn(Trt)-OH, Fmoc-Gaba-OH[2], Fmoc-His(Trt)-OH, , Fmoc-Gln(Trt)-OH, Fmoc-Ser(*t*Bu)-OH, Fmoc-Val-OH, Fmoc-Trp(Boc)-OH, Fmoc-Tyr(tBu)-OH, Fmoc-Ser(PO(OH)(OBn))-OH, Fmoc-Thr(PO(OH)(OBn))-OH, Fmoc-Tyr(PO(OH)(OBn))-OH and Fmoc-Tyr(PO(NMe$_2$)$_2$)-OH. Q-Sepharose FF resin was purchased from GE Healthcare. Other reagents were purchased from Sigma Aldrich, Alfa Aesar, Merck or Fisher Scientific and were used without further purification.

Peptides were synthesised manually using standard solid phase synthesis protocols:
Resin (amount specified in each reaction) was swollen in DMF (2-5 mL per 100 mg of resin – the same volume was used for the wash steps) for 30 min with agitation (Stuart Rotator SB2). The solution was then removed by vacuum filtration and the first coupling mixture added. Coupling reactions of commercially available amino acids were carried out using 5 equiv. (with respect to resin loading) Fmoc-amino acid, 4.9 equiv. HCTU and 10 equiv. DIPEA in DMF (2-5 mL per 100 mg of resin) for 60 minutes with agitation – unless otherwise stated. After the coupling reaction the solution was removed by filtration and the resin was washed with DMF (3 × 2 min), 20% piperidine in DMF (*v/v*, 5 × 2 min) and DMF (5 × 2 min). Subsequent couplings were carried out in the same manner.
Peptide N-termini were acetylated by mixing the resin with a solution containing acetic anhydride (5 equiv.) and DIPEA (5 equiv.) in DMF (2-5 mL per 100 mg of resin) for 30 min with mixing, the solution removed by vacuum filtration and the resin washed with DMF (3 × 2 min).

To prepare the resin for the cleavage reaction it was washed with DCM (3 × 2 min) and MeOH (3 × 2 min) then dried overnight *in vacuo*. The peptide was cleaved from the resin by mixing with a cleavage cocktail (2-5 mL per 100 mg of resin) consisting of TFA (95%), H_2O (2.5%) and TIS (2.5%). The cleavage cocktail was applied to the resin and mixed for 2 h, then dripped into cold ether (10 × volume of cleavage cocktail – same volume used in later ether washes) and the precipitate collected by centrifugation (4,000 × *g*, 5 minutes). The

[1] synthesised as described in reference 6 of the main article
[2] Gaba = γ-aminobutyric acid

ethereal supernatant was decanted, the peptide pellet resuspended in cold ether and centrifuged again. This was repeated at least 3 more times before residual ether was removed under a stream of nitrogen. The resultant amorphous solid was dissolved in the minimum volume H_2O (with the addition of small portions of MeCN/Dioxane as required), frozen and lyophilised.

Ion exchange chromatography was used to purify some peptides using the following method: lyophilised peptide was dissolved in H_2O and passed through a bed of Q-Sepharose FF resin (1 mL) which was washed with 1 M $NH_4HCO_{3(aq)}$ (10 mL) then H_2O (10 mL) prior to use under gravity. The peptide was then eluted with a stepwise gradient of $NH_4HCO_{3(aq)}$ (10, 20, 50, 100, 200 and 500 mM, 10 mL per elution). Fractions containing the peptide were identified by LCMS and combined (as appropriate) and lyophilised.

HPLC analysis was performed on an Agilent 1290 infinity LC using an Ascentis® Express Peptide ES-C18 column (10 cm × 2. 1 mm, 2.7 µM particle size) with a flow rate of 0.5 mL min^{-1} using a gradient of solvent B into solvent A. Solvent A – H_2O + 0.1% TFA, solvent B – MeCN + 0.1% TFA. The presence of compounds was monitored by the UV absorbance at 220, 230, 250 and 280 nm.

AcHN-Ser-pTyr-Val-Asn-Val-Gln-NH_2 11

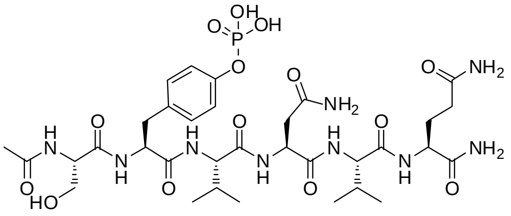

11

Peptide **11** was synthesised using Rink Amide Novagel™ resin (0.64 mmol/g loading; 100 mg, 0.064 mmol). Coupling of Fmoc-Tyr(PO(OH)(OBn))-OH was carried out using 5 equiv. amino acid, 4.9 equiv HATU and 10 equiv DIPEA in DMF for 1 hour. After cleavage and lyophilisation, the crude peptide was dissolved in H_2O and purified by ion-exchange chromatograhpy. After lyophilisation of relevant fractions, peptide **11** was obtained as a flocculent colourless solid (26 mg, 0.0313 mmol, 49% yield). *m/z*: Found *M(-H)* 828.3291, $C_{32}H_{51}N_9O_{14}P$ requires 828.3299 ; HPLC (5-95% B): retention time 1.26 min, 79%.

AcHN-Ser-phTz-Val-Asn-Val-Gln-NH_2 15

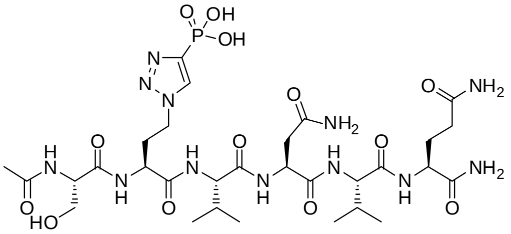

15

Peptide **15** was synthesised using Rink Amide Novagel™ resin (0.64 mmol/g loading; 100 mg, 0.064 mmol). Coupling of Fmoc-phTz(OBn)$_2$-OH **14** was carried out using 3 equiv. amino acid (125 mg, 0.192 mmol), 2.9 equiv. HCTU and 6 equiv. DIPEA in DMF for 1 hour. After cleavage and lyophilisation, the crude peptide was dissolved in H_2O and purified by ion-exchange chromatograhpy. After lyophilisation of relevant fractions, peptide **15** was obtained as a flocculent colourless solid (11 mg, 0.0134 mmol, 21% yield). *m/z*: Found *M(-H)* 817.3390, $C_{30}H_{50}N_{12}O_{13}P$ requires 817.3363; HPLC (5-95% B): retention time 1.12 min, 100%.

AcHN-Ser-pTz-Val-Asn-Val-Gln-NH_2 16

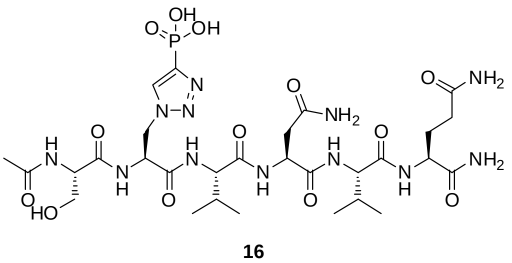

16

Peptide **16** was synthesised using Rink Amide Novagel™ resin (0.64 mmol/g loading; 100 mg, 0.064 mmol). Coupling of Fmoc-pTz(OBn)$_2$-OH was carried out using 3 equiv. amino acid (123 mg, 0.192 mmol), 2.9 equiv. HCTU and 6 equiv. DIPEA in DMF for 1 hour. After cleavage and lyophilisation, the crude peptide was dissolved in H_2O and purified by ion-exchange chromatograhpy. After lyophilisation of relevant fractions, peptide **16** was obtained as a flocculent colourless solid (6.81 mg,

0.0085 mmol, 13% yield). δ_H[3] (500 MHz, CD_3OD): 8.02 (1H, s, pTz-ArH), 5.01-4.91 (1H, m, Asn4-H_α), 4.41 (1H, t, $J_{\alpha\text{-}\beta}$ 5.6, Ser1-H_α), 4.34 (1H, dd, $J_{\alpha\text{-}\beta1}$ 9.5, $J_{\alpha\text{-}\beta2}$ 5.1, Gln6-H_α), 4.19-4.11 (2H, m, Val3-H_α & Val5-H_α), 3.77 (2H, d, $J_{\beta\text{-}\alpha}$ 5.9, Ser1-H_β), 2.94-2.78 (2H, m, Asn4-H_β), 2.45-2.39 (2H, m, Gln6-H_γ), 2.33-2.11 (2H, m, Val3-H_β & Val5-H_β), 1.01-0.91 (12H, m, Val3-H_γ & Val5-H_γ), 2.10 (3H, s, Ac), 2.09-2.09 (2H, m, Gln6-H_β).*m/z*: Found *M(-H)* 803.3241, $C_{29}H_{48}N_{12}O_{13}P$ requires 803.3207; HPLC (5-95% B): retention time 1.12 min, 92%.

FITC-Gaba-Ser-pTyr-Val-Asn-Val-Gln-NH_2 17

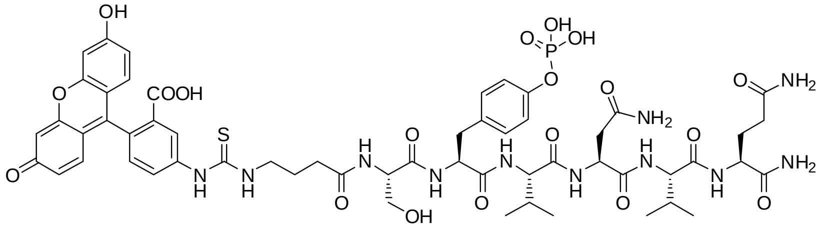

17

Peptide **17** was synthesised using Rink Amide Novagel™ resin (0.64 mmol/g loading; 100 mg, 0.064 mmol). Coupling of Fmoc-Tyr($PO(NMe_2)_2$)-OH was carried out using 5 equiv. amino acid, 5 equiv. HCTU and 10 equiv. DIPEA in DMF for 1 hour. The cleavage cocktail was applied to the resin and mixed for 2 h, 10% H_2O was added and the resin was swelled overnight. The peptide was then precipitated according to standard procedure and lyophilised, the crude peptide was dissolved in H_2O and purified by ion-exchange chromatography. After lyophilisation of relevant fractions, peptide **17** was obtained as a flocculent orange solid (46 mg, 0.0364 mmol, 57% yield). *m/z*: Found *M(-2H)* 629.6998, $C_{56}H_{66}N_{11}O_{19}PS$ requires 629.7003; HPLC (5-95% B): retention time 2.16 min, 100%.

AcHN-Ser-Tyr-Val-Asn-Val-Gln-NH_2 18

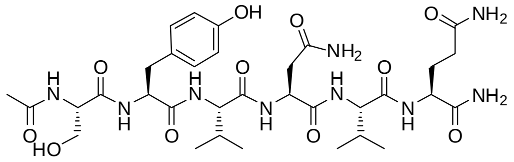

18

Peptide 18 was synthesised using Rink Amide Novagel™ resin (0.64 mmol/g loading; 100 mg, 0.064 mmol). After cleavage and lyophilisation, the crude peptide was dissolved in H_2O and purified by ion-exchange chromatography. After lyophilisation of relevant fractions, peptide **18** was obtained as a flocculent colourless solid (32 mg, 0.0427 mmol, 67% yield). *m/z*: Found *M(+H)* 750.3787, $C_{33}H_{52}N_9O_{11}$ requires 750.3781; HPLC (5-95% B): retention time 1.20 min, 67%.

AcHN-Ser-His-Val-Asn-Val-Gln-NH_2 19

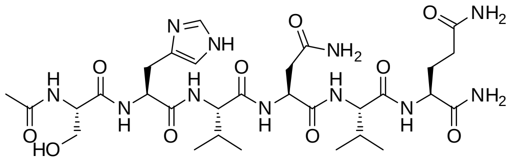

19

Peptide **19** was synthesised using Rink Amide Novagel™ resin (0.67 mmol/g loading; 144 mg, 0.1005 mmol). After cleavage and lyophilisation, the crude peptide was dissolved in H_2O and purified by ion-exchange chromatography (using SP-Sepharose FF instead of Q-Sepharose FF). After lyophilisation of relevant fractions, peptide **19** was obtained as a flocculent colourless solid (25 mg, 0.0345 mmol, 34%). *m/z*: Found *M(+H)* 724.3746, $C_{30}H_{50}N_{11}O_{10}$ requires 724.3737; HPLC (5-95% B): retention time 0.81 min, 73%.

[3] Signals for pTz-Hα & Hβ are coincident with the suppressed residual water peak.

AcHN-Ser-pSer-Val-Asn-Val-Gln-NH_2 20

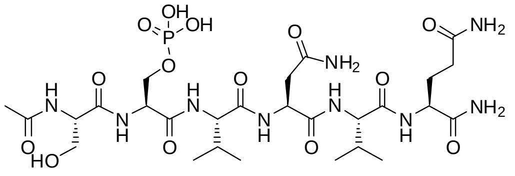

20

Peptide **20** was synthesised using Rink Amide Novagel™ resin (0.67 mmol/g loading; 144 mg, 0.1005 mmol). Coupling of Fmoc-Ser(PO(OH)(OBn))-OH was carried out using 5 equiv. amino acid, 5 equiv HCTU and 10 equiv DIPEA in DMF for 1 hour. After cleavage and lyophilisation, the crude peptide was dissolved in H_2O and purified by ion-exchange chromatograhpy. After lyophilisation of relevant fractions, peptide **20** was obtained as flocculent colourless solid (35 mg, 0.0464 mmol, 46%). Found *M(+2Na)* 421.6240, $C_{27}H_{46}N_9Na_4O_{14}P$ requires 421.6241; HPLC (5-95% B): retention time 0.74 min, 51%.

AcHN-Ser-pThr-Val-Asn-Val-Gln-NH_2 21

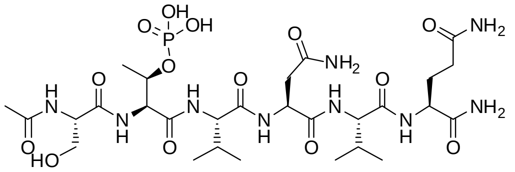

21

Peptide **21** was synthesised using Rink Amide Novagel™ resin (0.67 mmol/g loading; 144 mg, 0.1005 mmol). Coupling of Fmoc-Thr(PO(OH)(OBn))-OH was carried out using 5 equiv. amino acid, 5 equiv. HCTU and 10 equiv. DIPEA in DMF for 1 hour. After cleavage and lyophilisation, the crude peptide was dissolved in H_2O and purified by ion-exchange chromatography. After lyophilisation of relevant fractions, peptide **21** was obtained as flocculent colourless solid (28 mg, 0.0364, 36%) Found *M(-H)* 766.3159, $C_{28}H_{49}N_9O_{14}P$ requires 766.3142; HPLC (5-95% B): retention time 0.76 min, 53%.

H-Cys-Gly-Ala-Gly-Ala-Gly pTyr-Gly-Ala-Gly-Ala-Gly-OH S1

S1

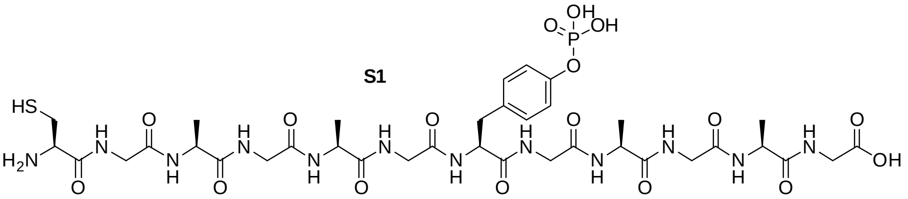

Peptide **S1** was synthesised using chlorotrityl resin preloaded with glycine (0.64 mmol/g loading; 200 mg, 0.128 mmol). Coupling of Fmoc- Tyr(PO(OH)(OBn))-OH was carried out using a double coupling procedure using first 1 equiv. amino acid, 1 equiv. HATU and 2 equiv. DIPEA in DMF for 1 hour, then using 2 equiv. amino acid, 2 equiv HATU and 4 equiv DIPEA in DMF for 1 hour. Following lyophilisation, peptide **S1** was obtained as a flocculent colourless solid (93 mg, 0.093 mmol, 73% yield). δ_H (300 MHz, D_2O): 7.13 (2H, d, $^3J_{H\text{-}H}$ 8.5, pTyr- 2 × ArH_2), 7.06 (2H, d, $^3J_{H\text{-}H}$ 8.5, pTyr- 2 × ArH_3), 4.56-4.45 (1H, m, pTyr- C*H*CH_2Ar), 4.35-4.17 (5H, m, 4 × Ala-C*H*CH_3 + Cys- C*H*CH_2SH), 3.99-3.70 (12H, 6 × Gly- CH_2), 3.12-3.03 (1H, m, pTyr - CHC*H*HAr), 3.12-2.98 (2H, m, Cys- CHCH_2SH), 2.98-2.85 (1H, m, pTyr- CHCH*H*Ar), 1.39-1.29 (12H, m, 4 × Ala-CHCH_3); δ_P(121 MHz, D_2O): -4.14 (s); *m/z*: Found *M(-H)* 989.3241, $C_{36}H_{54}$ $N_{12}O_{17}PS$ requires 989.3194.

H-Cys-Gly-Ala-Gly-Ala-Gly pTz-Gly-Ala-Gly-Ala-Gly-OH S2

S2

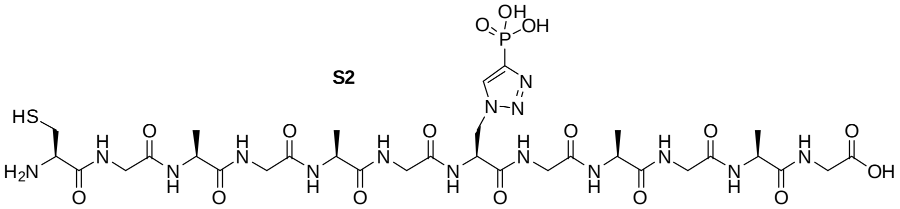

Peptide **S2** was synthesised as previously described – see reference 7 of the main article.

Protein expression and purification

Mass Spectrometry was performed on a Bruker HCT Ultra ion trap mass spectrometer

GST-Grb2

The cDNA for hGrb2 gene in a pGEX-2T vector (a kind gift from Dr Andrew MacDonald - University of Leeds) was transformed into *E. coli* BL21-Gold (DE3) cells which were grown in a 1 L culture of auto-inducing media (AIM)[4] at 37 °C with agitation for 24 h. The cells were collected by centrifugation (10,000 × *g*, 10 minutes) and resuspended in ~50 mL of lysis buffer 1 (50 mM NaH_2PO_4/Na_2HPO_4, 120 mM NaCl, 10 mM EDTA, pH 7.4 at 4 °C). The cells were then lysed in a constant cell disrupter (20 psi), the insoluble fraction collected by centrifugation (35,000 × *g*, 45 minutes) and the resultant supernatant loaded onto a GSTrap HF column connected to an Äkta Purifier FPLC. The column was washed with 5 column volumes (CV) of lysis buffer 1 then a gradient (0-100%) of elution buffer 1 (lysis buffer 1 + 10 mM glutathione) over 3 CV then 5 CV 100% elution buffer 1 while collecting fractions and monitoring absorbance at 280 nm (Figure S1-A).

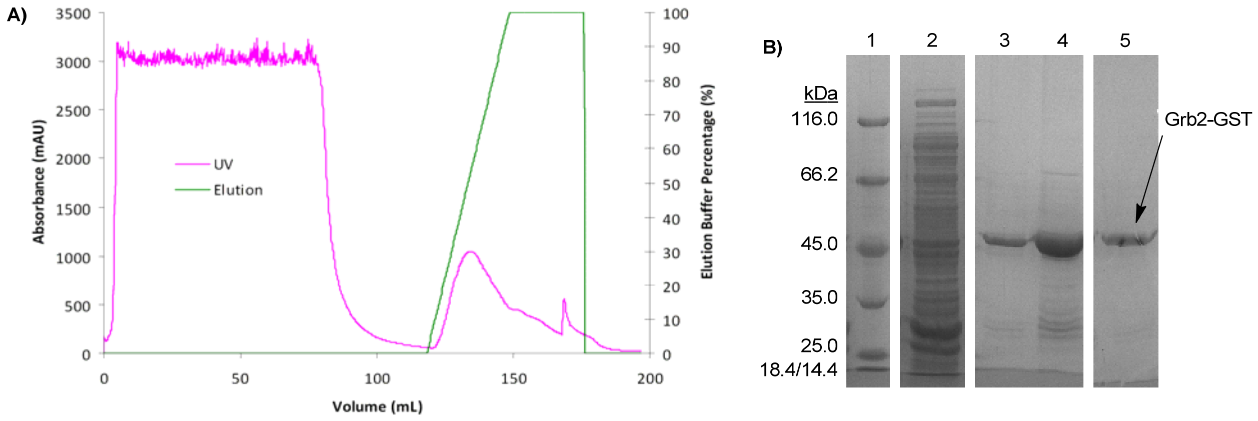

Figure S1. A) Affinity purification by GSTrap HF column. B) 10% SDS-PAGE of fractions: lane 1 contains MW markers while lanes 2-5 contain samples at 50, 120, 130 and 180 mL respectively.

Fractions containing the protein were analysed by SDS-PAGE (Figure S1), the relevant fractions combined, concentrated (using an Amicon Ultra centrifugal concentrator with a 30,000 MW cut-off) to a total volume of < 1 mL and further purified by size-exclusion chromatography (SEC) on a Superdex 200 10/300 column equilibrated and eluted with ITC buffer (50 mM Tris, 100 mM NaCl, pH 7.4 at 25°C). Fractions containing the protein were identified by SDS-PAGE (Figure S2), combined and concentrated (as before) and the protein concentration determined by UV absorbance.

GST-Grb2, 52014 Da, $\varepsilon = 81165\ M^{-1}\ cm^{-1}$:
MSPILGYWKIKGLVQPTRLLLEYLEEKYEEHLYERDEGDKWRNKKFELGLEFPNLPYYIDGDVKLTQSMAIIRYIADKHNMLGGCP
KERAEISMLEGAVLDIRYGVSRIAYSKDFETLKVDFLSKLPEMLKMFEDRLCHKTYLNGDHVTHPDFMLYDALDVVLYMDPMCL
DAFPKLVCFKKRIEAIPQIDKYLKSSKYIAWPLQGWQATFGGGDHPPKLVPRGSMEAIAKYDFKATADDELSFKRGDILKVLNEEC
DQNWYKAELNGKDGFIPKNYIEMKPHP*WFFGKIPRAKAEEMLSKQRHDGAFLIRESESAPGDFSLSVKFGNDVQHFKVLRDGAGKYFL*
*WVVKFNSLNELVDYHRSTSVSRNQQIFLRDIE*QVPQQPTYVQALFDFDPQEDGELGFRRGDFIHVMDNSDPNWWKGACHGQTGMFPR
NYVTPVNRN*V*GFKPNSS
[Grb2 is shown underlined with the SH2 domain italicised]

---

[4] Auto-Inducing Media components: 50% (*w/v*) glucose solution and 25% (*w/v*) lactose solution were filter sterilised before use; 50% (*v/v*) glycerol; 5000× Trace Metals (50 mM $FeCl_3$, 20 mM $CaCl_2$, 10 mM $MnCl_2$, 2 mM $ZnSO_4$, 2 mM $CoCl_2$, 2 mM $CuCl_2$, 2 mM Na_2MoO_4, 2 mM Na_2SeO_3, 2 mM H_3BO_3, 2 mM $NiCl_2$, 60 mM HCl); 50× Salts (1.25 M Na_2HPO_4, 1.25 M KH_2PO_4, 2.5 M NH_4Cl, 1.25 M Na_2SO_4).

Recipe: Tryptone Plus 10 g, Yeast Extract 5 g, 50× salts 20 mL, 50% glycerol 10 mL, 2M $MgCl_2$ 1mL, 5000× Metals 200 µL. This mixture was then made up to 1L with water, autoclaved and allowed to cool before 50% glucose 1 mL and 25% lactose 8 mL were added.

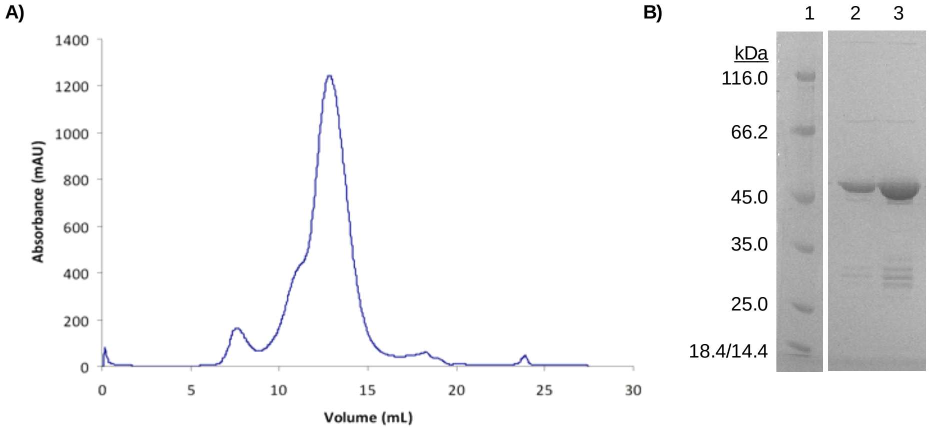

Figure S2. *A) SEC using Superdex 200 10/300 column eluting with ITC buffer. B) 10% SDS-PAGE of fractions: lane 1 contains MW markers while lanes 2 & 3 contain samples at 10 and 13 mL respectively.*

His_6-(Grb2-SH2)

The SH2 domain was subcloned out of the pGEX-2T plasmid using primers designed amplify the relevant part of the gene (which was subsequently purified by agarose gel electrophoresis and extracted) and allow ligation into a pET-28a plasmid between the BamH1 and Nde1 restriction sites. After ligation, the sequence of this resultant plasmid was verified by DNA sequencing and used to transform *E. coli* C41 (DE3) cells, which were grown in a 1 L culture of AIM supplemented with 100 mg/L ampicillin at 37 °C with agitation for 24 h. The cells were collected by centrifugation (10,000 × *g*, 10 minutes) and resuspended in ~50 mL of lysis buffer 2 (50 mM NaH_2PO_4/Na_2HPO_4, 300 mM NaCl, pH 7.4 at 4 °C). The cells were then lysed in a constant cell disrupter (20 psi), the insoluble fraction was collected by centrifugation (35,000 × *g*, 45 minutes). The supernatant was subjected to purification on Ni-NTA agarose resin (from Qiagen using the protocol described below), but this did not yield the expected His_6-(Grb2-SH2) (Figure S3 A).

The cellular debris from cell lysis was resuspended in ~ 50 mL wash buffer 1 (20 mM Tris, 200 mM NaCl, 2 mM EDTA, 1.5% (*v/v*) Triton X-100, pH 8.0 at 25 °C) and stirred at room temperature for 30 minutes. The mixture was centrifuged (17,000 × *g*, 20 minutes), the supernatant decanted and the insoluble fraction resuspended in ~50 mL wash buffer 2 (10 mM Tris, 1000 mM NaCl, 1 mM EDTA, 1.5% (*v/v*) Triton X-100, pH 8.0 at 25 °C). This was stirred at room temperature for 20 min, centrifuged (17,000 × *g*, 20 minutes) and the supernatant decanted. Washing with wash buffer 2 was repeated twice more and the insoluble fraction suspended in ~50 mL solubulisation buffer (lysis buffer 2 + 8M Urea) by stirring at room temperature overnight then centrifuged (17,000 × *g*, 20 minutes) to remove insoluble material.

The solubulised protein was loaded onto a column of Ni-NTA resin (pre-equilibrated in lysis buffer 2) under gravity and the flow-through collected. The column was washed with lysis buffer 2 (5 CV), lysis buffer 2 + 10 mM imidazole (5 CV) and lysis buffer 2 + 50 mM imidazole (5 CV) before being eluted with elution buffer 2 (lysis buffer 2 + 250 mM imidazole, 5CV). The collected flow through was then reapplied to the resin and the above procedure repeated. The fractions containing the desired protein were identified by SDS-PAGE (Figure S3-B) and the relevant fractions combined and concentrated (Amicon Ultra centrifugal concentrator, 10,000 MW cut-off) to < 2 mL.

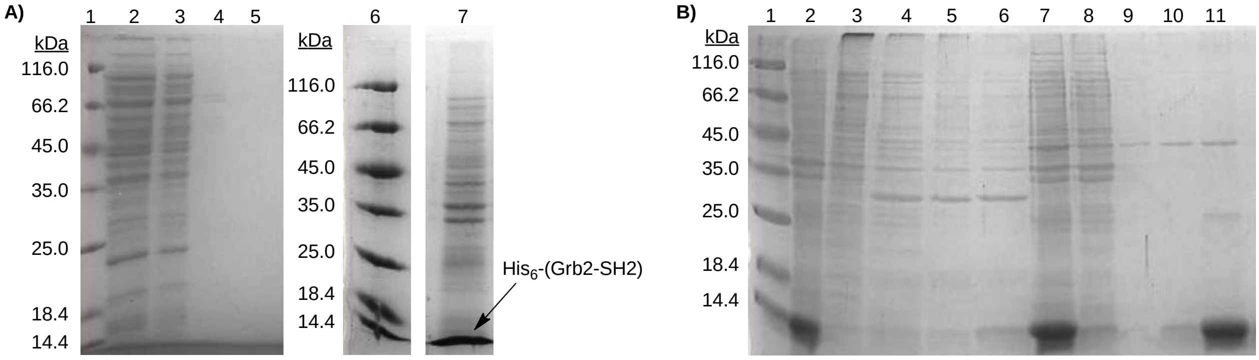

Figure S3. *A)15% SDS-PAGE: Lane 1 - MW markers, 2 & 3 –flow through Ni-NTA column of soluble fraction, 4 & 5 – elution of soluble fraction from Ni-NTA column, 6 – MW markers, 7 – insoluble fraction. B) 15% SDS-PAGE: Lane 1 - MW markers, 2 - insoluble fraction, 3 – superntant of wash with wash buffer 1, 4-6 - superntants of washes with wash buffer 2, 7 – protein solubulised in 8 M urea, 8 – flow through of solubilised protein, 9 – wash with 10 mM imidazole, 10 – wash with 50 mM imidazole, 11 – elution with 250 mM imidazole.*

The protein was further purified by SEC using a Superdex 75 26/60 column equilibrated and eluted with 50 mM Tris, 100 mM NaCl pH 7.4 (ITC buffer). This showed two distinct peaks, which both appeared to be His_6-(Grb2-SH2) by SDS-PAGE and ESI-mass spectrometry (Figure S4). ITC with pY peptide 11 revealed that the earlier peak, peak 1 (suggesting a larger protein/protein complex than the later peak), had a significantly reduced affinity for the peptide compared with the later peak, peak 2. Consultation of the literature (ref 20, main article) revealed that the Grb2-SH2 domain can form a domain-swapped dimer which has a reduced affinity for their control peptide; in this case tripeptide AcHN-pTyr-Val-Asn-NH_2 which itself has ~5-fold lower affinity for monomeric Grb2-SH2 than our chosen hexapeptide **11** (K_d = 1.6 μM *c.f.* 0.34 μM, see later). Protein from peak 2 was used in all subsequent experiments.

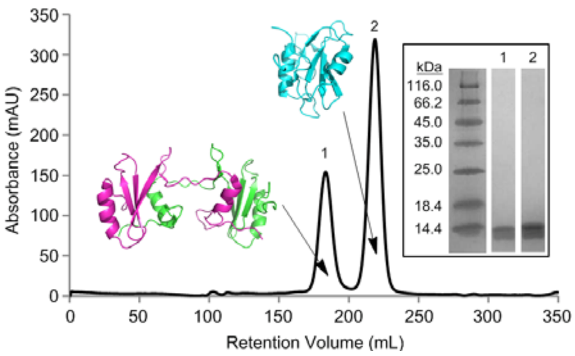

***Figure S4.**. SEC trace of affinity-purified His*$_6$*-(Grb2-SH2) using a Superdex 75 26/60 column equilibrated and eluted with ITC buffer. Both peaks were identified to contain the same monomeric protein unit with a mass corresponding to His*$_6$*-(Grb2-SH2) as shown by SDS-PAGE [15%, inset] and mass-spectrometry. Peak 1 gave a mass of 12998.86 Da, while peak 2 gave a mass of 12999.26 Da; His*$_6$*-(Grb2-SH2) should have a mass of 13000.6 Da (following removal of the N-terminal methionine, see below). ITC with peptide **11** revealed peak 1 had a ~14 fold lower affinity than peak 2 (*K_d = 4.8 μM *c.f.* 0.34 μM, *Figure S8).*

His_6-(Grb2-SH2), 13132 Da, ε = 13980 M^{-1} cm^{-1}:
MGSSHHHHHHSSGLVPRGSHMWFFGKIPRAKAEEMLSKQRHDGAFLIRESESAPGDFSLSVKFGNDVQHFKVLRDGAGKYFLWV
VKFNSLNELVDYHRSTSVSRNQQIFLRDIE

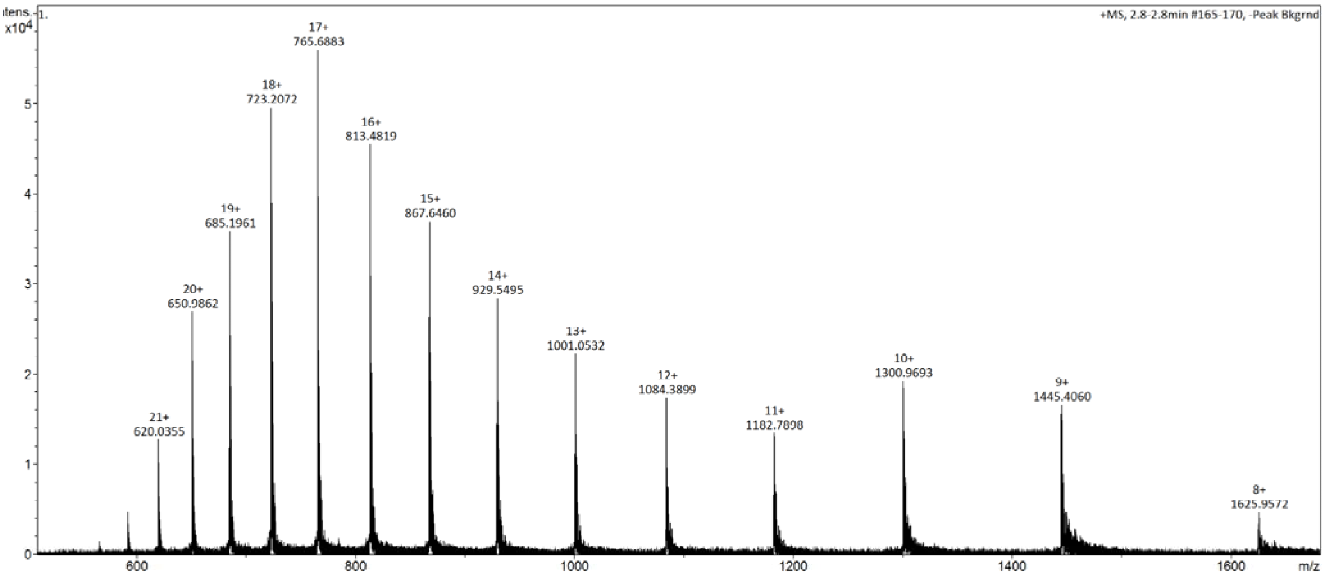

Figure S5: *ESMS trace for Grb2-SH2 – measured mass 12999.8 ± 0.1 kDa consistent with cleavage of the N-terminal methionine (expected mass 13000.6)*

Isothermal Titration Calorimetry (ITC)

ITC experiments were carried out using a Microcal VP-ITC or Microcal ITC200. Protein samples were concentrated using Amicon Ultra centrifugal concentrators. Concentrations were calculated using the Beer-Lambert equation: A = εcl (ε = extinction coefficient (M^{-1} cm^{-1}) calculated for each protein from the amino acid sequence using Expasy ProtParam (http://web.expasy.org/protparam/), c = concentration (M), l = path length (cm)). Ligand (injectant) was dissolved in buffer that had passed through the concentrator to ensure an exact buffer match. Samples were loaded into the cell using a gas-tight syringe and gently mixed to expel any bubbles. The injectant syringe was loaded with ligand of known concentration. Experiments were conducted at 25 °C, with a stirring speed of 1000 rpm.

Data were fitted using origin 7 with the Microcal ITC plug-in to a one-site binding model. First the area above each injection peak was calculated to give a value for the heat released upon binding of the peptide supplied by each injection. These data were then fitted using the Wiseman isotherm which relates the stepwise change in heat of the system normalised with respect to the moles of ligand added per injection ($dQ/d[X]_t$), to the absolute ratio of ligand to receptor concentration ($X_R = [X]_t/[M]_t$)at any point during the course of the titration (Figure S5). When the receptor was saturated by the end of the experiment (indicated by the isotherm values becoming ~constant) a fixed value for the heat of dilution was subtracted from the data. Otherwise a dilution experiment was carried out by injecting the ligand into the cell containing only buffer and no protein. These data were then fitted linearly and subtracted from the relevant experiment. The first data point was discraded prior to fitting in all cases.

$$\frac{dQ}{d[X]_t} = \Delta H^\circ V_0 \left[\frac{1}{2} + \frac{1 - X_R - r}{2\sqrt{(1 + X_R + r)^2 - 4X_R}} \right] \quad (1)$$

$$\frac{1}{r} = c = K_a[M]_t = \frac{[M]_t}{K_d} \quad (2)$$

***Figure S6.**. Wiseman isotherm.*

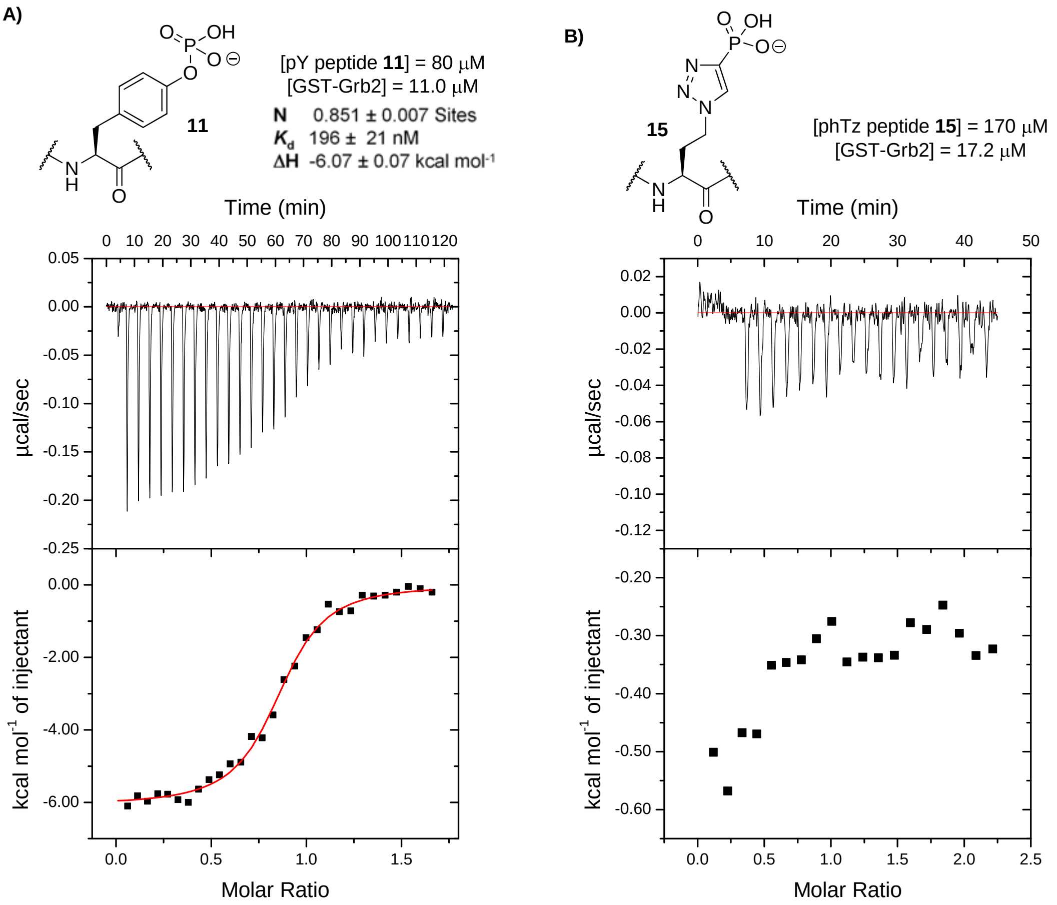

Figure S7. A) ITC trace of pY peptide **11** into GST-Grb2. B) ITC trace phTz peptide **15** into GST-Grb2

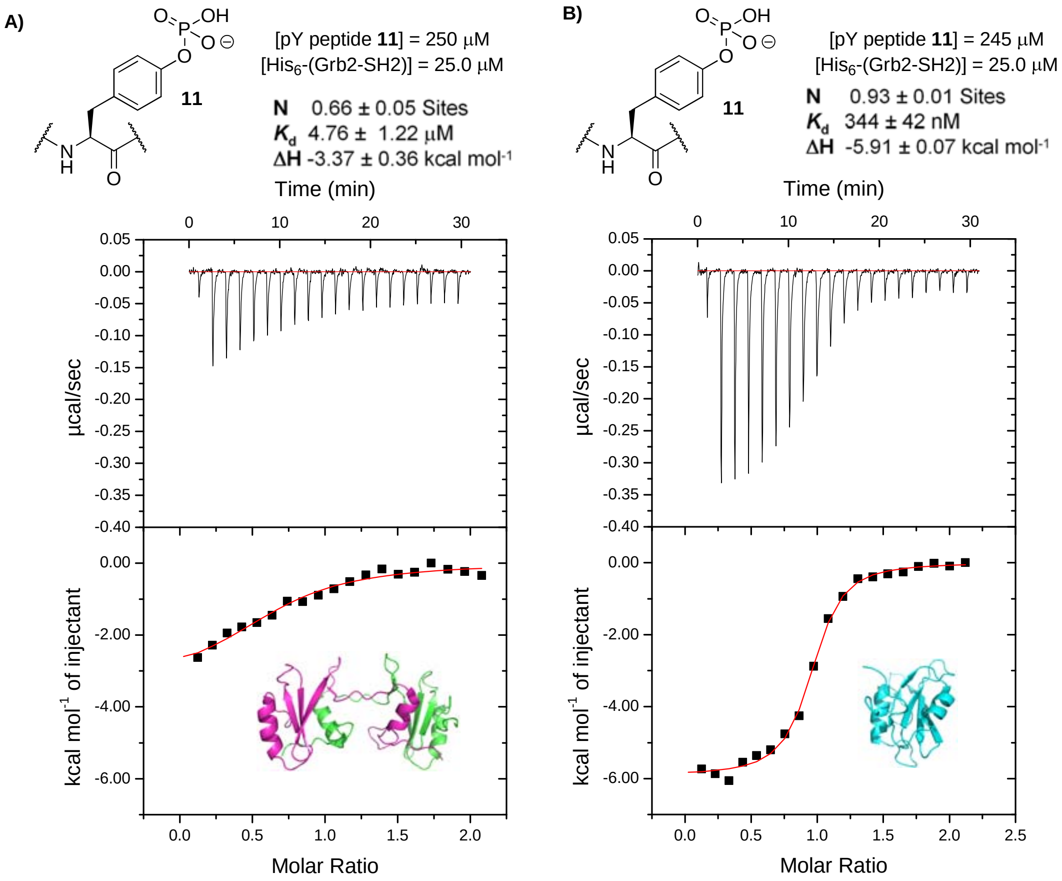

Figure S8. A) ITC trace of pY peptide **11** into the early-eluting fraction (peak 1, Figure S4) of His_6-(Grb2-SH2). B) ITC trace of pY peptide **11** into the late-eluting fraction (peak 2, Figure S4) of His_6-(Grb2-SH2). These results were used to establish the structures of the proteins eluting at different volumes.

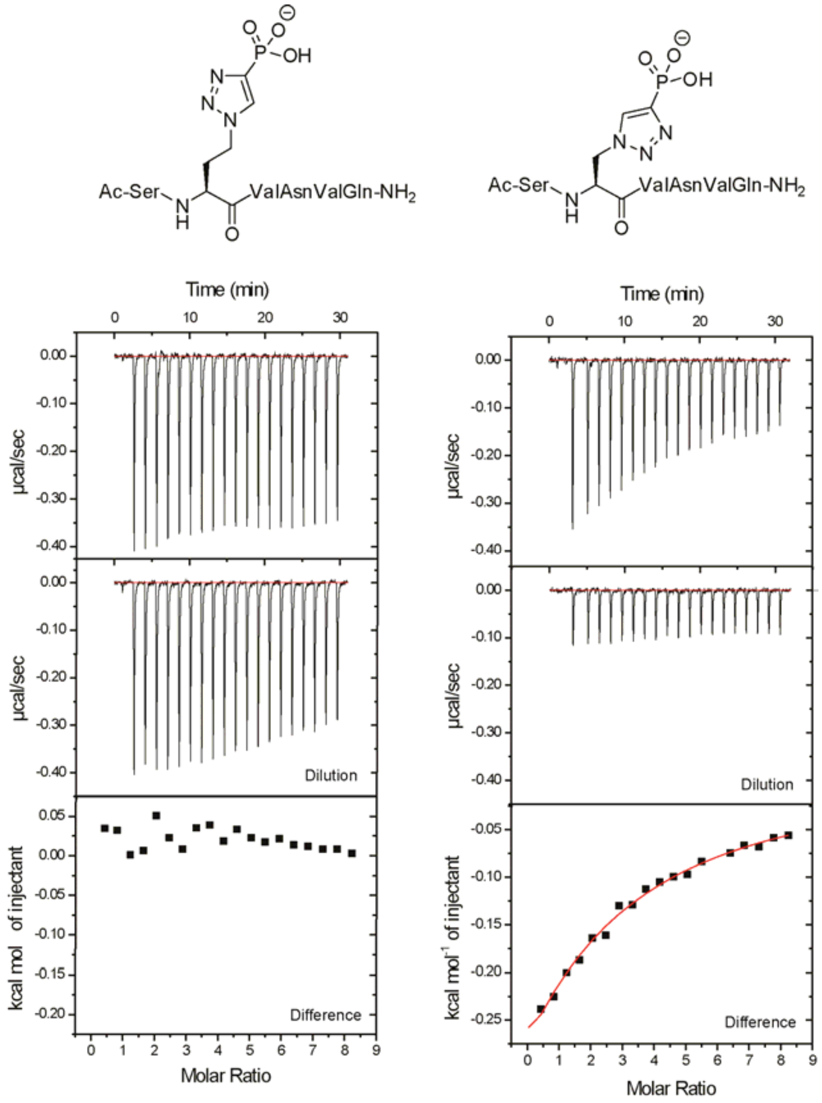

Figure S9. Control experiments for peptides **15** and **16** interacting with His_6-(Grb2-SH2). **Top:** titration of peptide 16 (right) and 15 (right) into Grb2-SH2**. Middle**: control titration of the same concentration of peptides into buffer. **Bottom**: Difference between titrations.

pKa Determination

NMR data were collected using a Bruker DPX300 and analysed using MestReNova software. Experiments were carried out 121 MHz using peptide **S1** or **S2** at a concentration of 10 mM in 10% D_2O in H_2O (*v*/*v*, 2 mL) with an external standard of *tris*-carboxyethyl phosphine (TCEP) in H_2O with a δ value set at 16.30 ppm.[5] The pH of the solution was measured and adjusted by addition of 100 mM NaOH in 10% D_2O in H_2O (*v*/*v*) in portions of 5 μL. At higher pH values significant signal-broadening was observed, which precluded accurate determination of chemical shifts and such results were not included in calculations to determine pKa's. The pKa was determined by fitting the observed chemical shift data to a Boltzmann distribution yielding pKas of ~5.8 for pTyr and 5.95 for the phosphotriazole analogue.

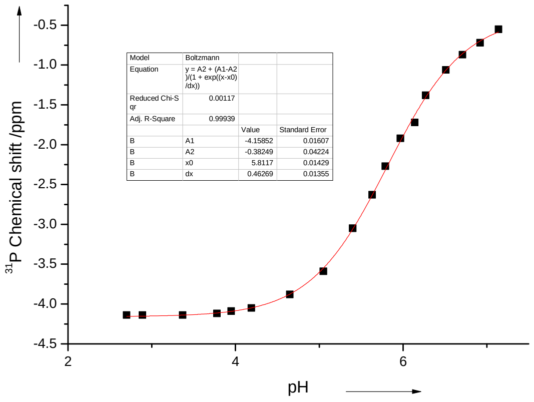

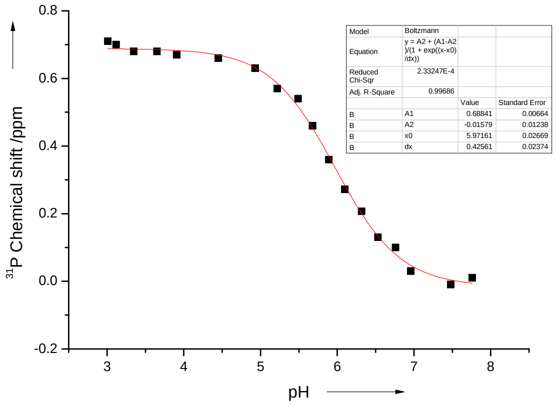

Figure S10. pH titrations of peptides **S1** and **S2** containing phosphotyrosine (**S1**, left) and phosphonotriazole (**S2**, right)

[5]Determined by a preliminary experiment using TCEP with an external standard of phosphoric acid (with a shift assigned as 0.00 ppm).

Fluorescence polarization experiments

Peptides were prepared as described above. Fluorescence polarization assays were carried out in 96 well plates (160 µL per well). All experiments were performed in 50 mM Tris.HCl, 100 mM NaCl pH 7.4 (ITC buffer). 2.5-fold or 3-fold dilution series of each peptide were routinely prepared before mixing in the plate well before data acquisition using a Perkin Elmer EnVision™ 2103 MultiLabel plate reader

*Determination of binding of FITC-pY peptide **17** to Grb2-SH2 domain*

Grb2-SH2 was serially diluted through ITC buffer containing 100 nM peptide **17** in triplicate and the fluorescence measured in parallel and perpendicular channels. The instrumental *G* factor for each experiment was approximated by assuming the anisotropy r = 0 for the free peptide. For each data point the total intensity I and anisotropy r were then determined by application of equations 3 and 4 below. Data were fit using a logistic model to obtain limiting maximum and minimum values for anisotropy and fluorescence intensity (see figure S11) Substantial fluorescence quenching was observed for the peptide upon binding of the protein and the fraction bound was therefore calculated using equation 5 where $\lambda = I_{bound}/I_{free}$. The fraction bound was then fit with a simple binding model to yield an approximate value for the K_d of 283 nM (figure S11).

$$I_T = I_{parallel} + 2GI_{perpendicular} \qquad (3)$$

$$r = \frac{I_{parallel} - GI_{perpendicular}}{I_T} \qquad (4)$$

$$F = \frac{r - r_{\min}}{R(r_{\max} - r) - r - r_{\min}} \text{ where } R = \frac{I_{bound}}{I_{free}} \qquad (5)$$

Competition binding assay

For competition assays, serial dilutions of peptides **11**, **15**-**16** & **18**-**21** were prepared together with 200 nM **17** and 175 nM Grb2-SH2. For those compounds showing a binding response (**11** & **16**) the anisotropy was converted to fraction bound before fitting using a logistic model to obtain an IC_{50} value.

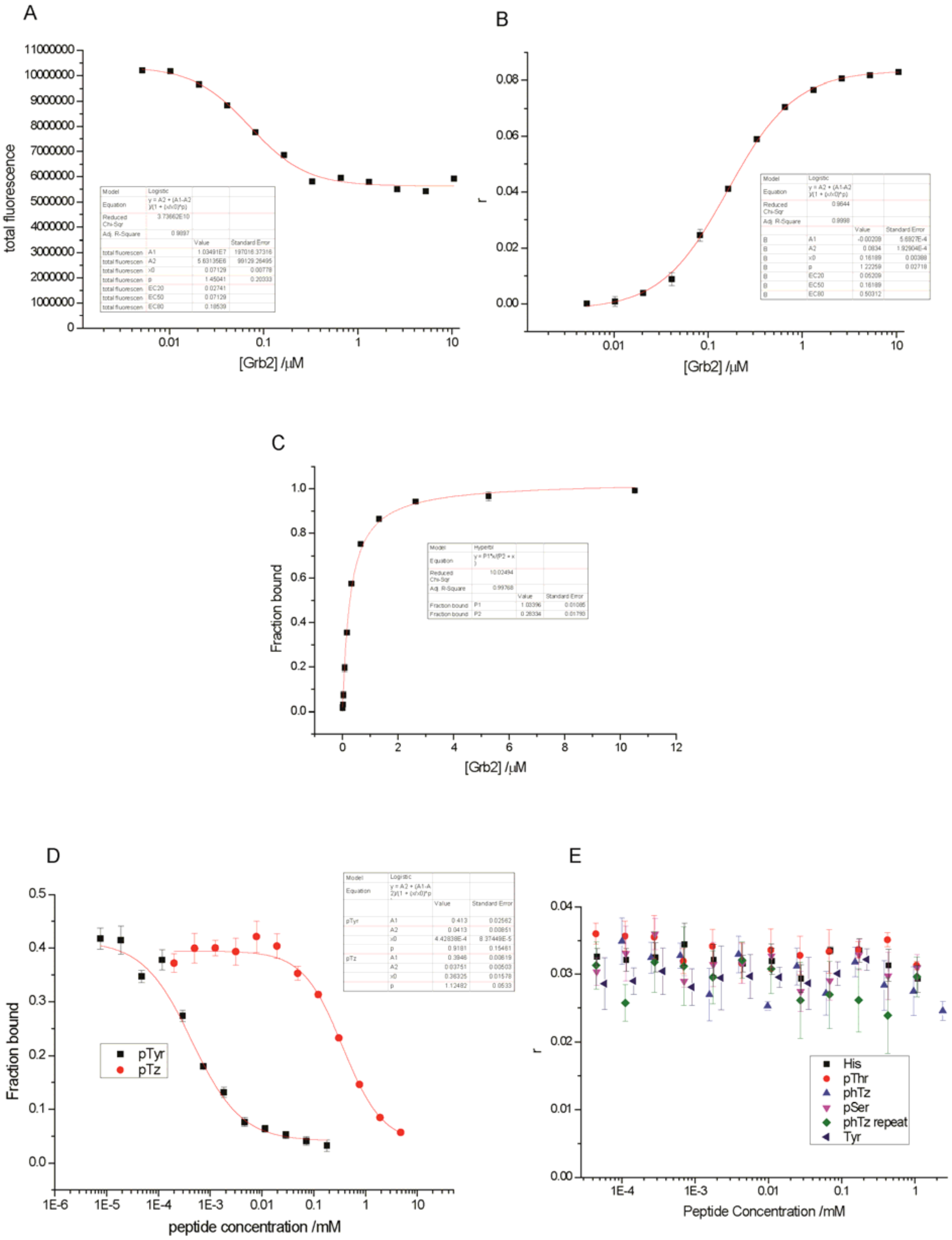

Figure S11 Fluorescence anisotropy analysis of peptides binding to the His_6-Grb2-SH2 domain A Concentration of fluorescence quenching as a function of [Grb2] with 100 nM FITC-labelled **17**. B. Dependence of anisotropy r upon [Grb2] C. Fraction bound peptide **17** as a function of [Grb2] D.Competition of peptides **11** (pTyr) and **16** (pTz) for binding to Grb2 over peptide **17**. E. No competition is evident for peptides **15**, **18**, **19**, **20** or **21**.

HPLC data

AcHN-Ser-pTyr-Val-Asn-Val-Gln-NH$_2$ 11

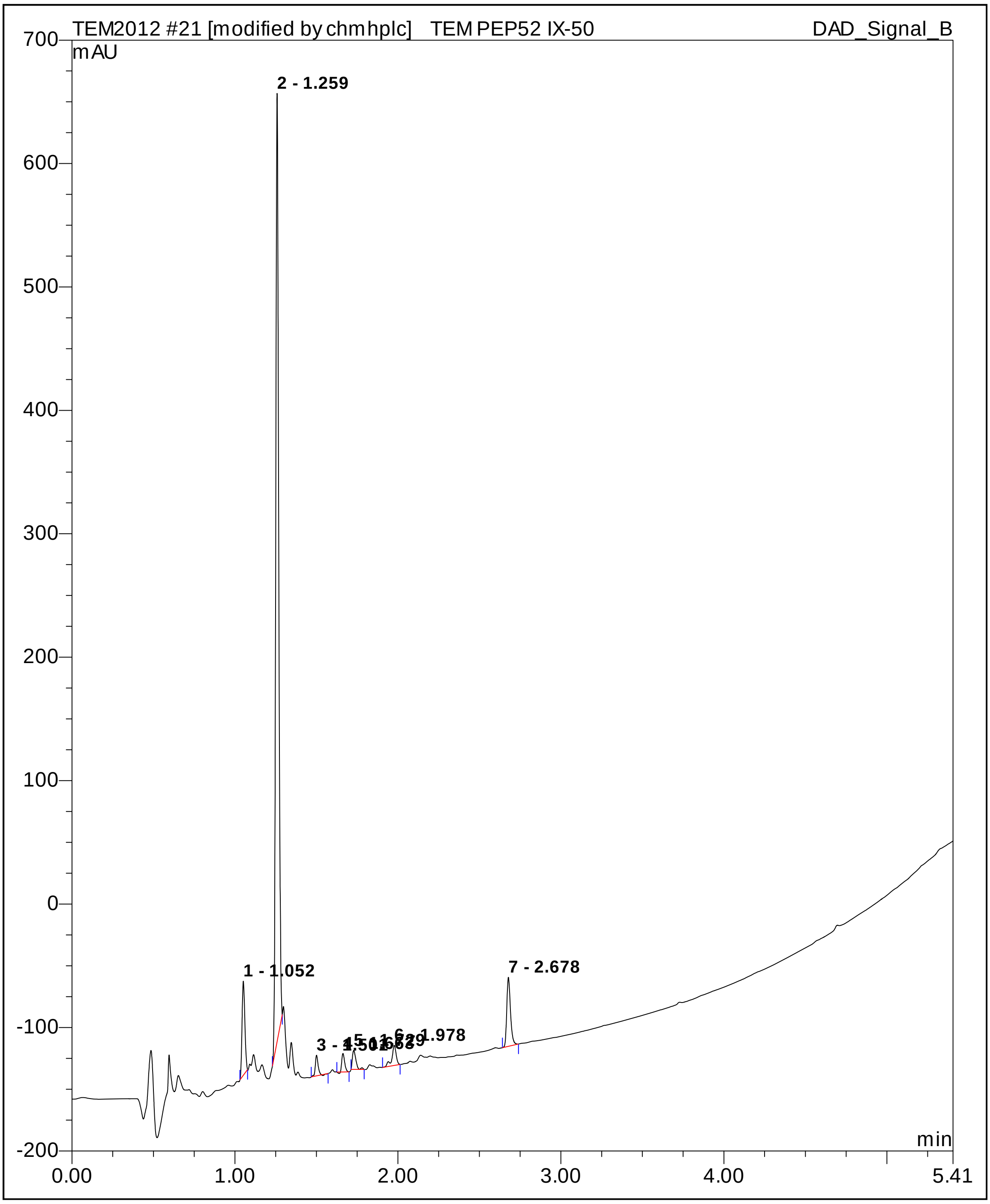

AcHN-Ser-phTz-Val-Asn-Val-Gln-NH_2 15

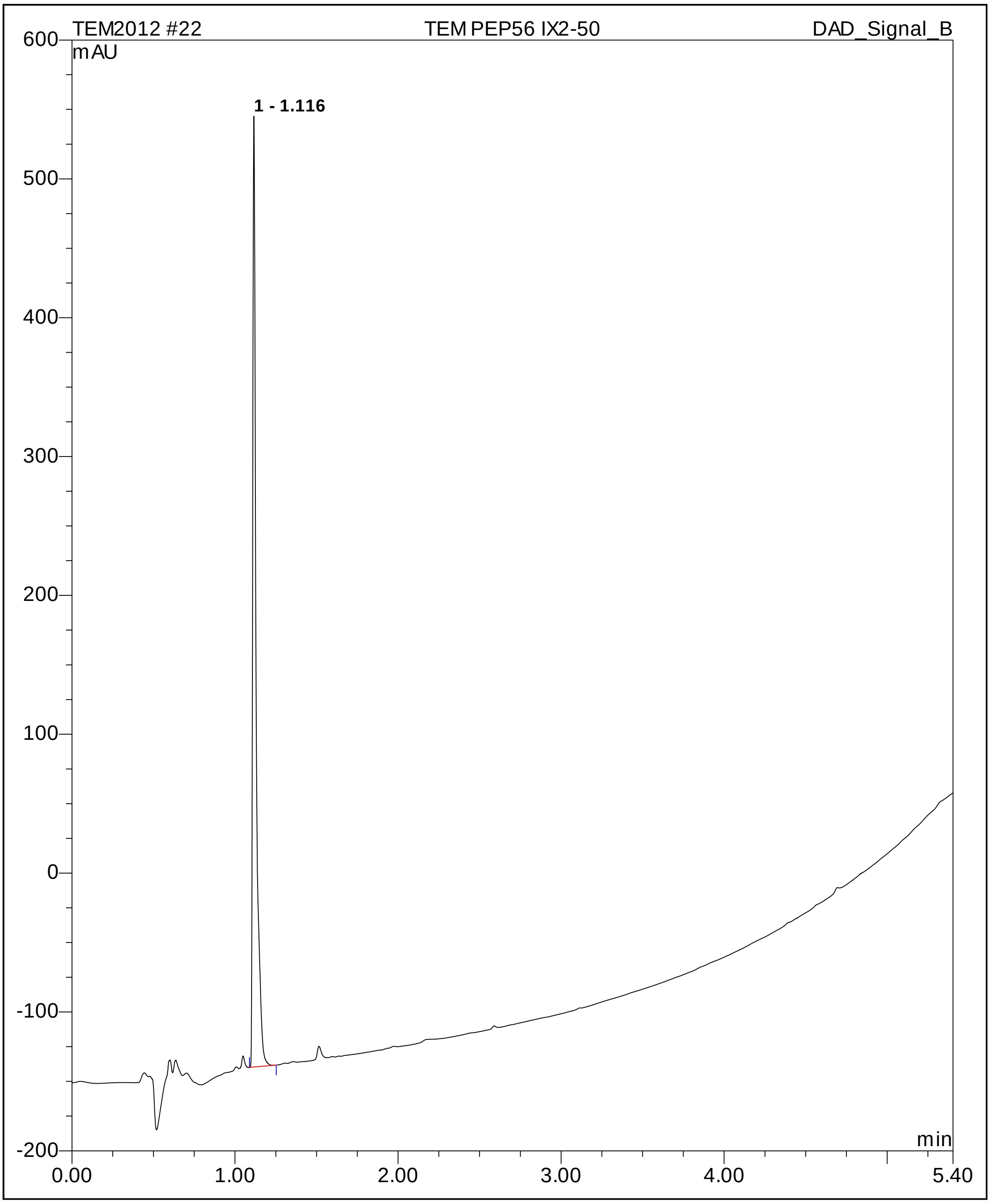

AcHN-Ser-pTz-Val-Asn-Val-Gln-NH_2 16

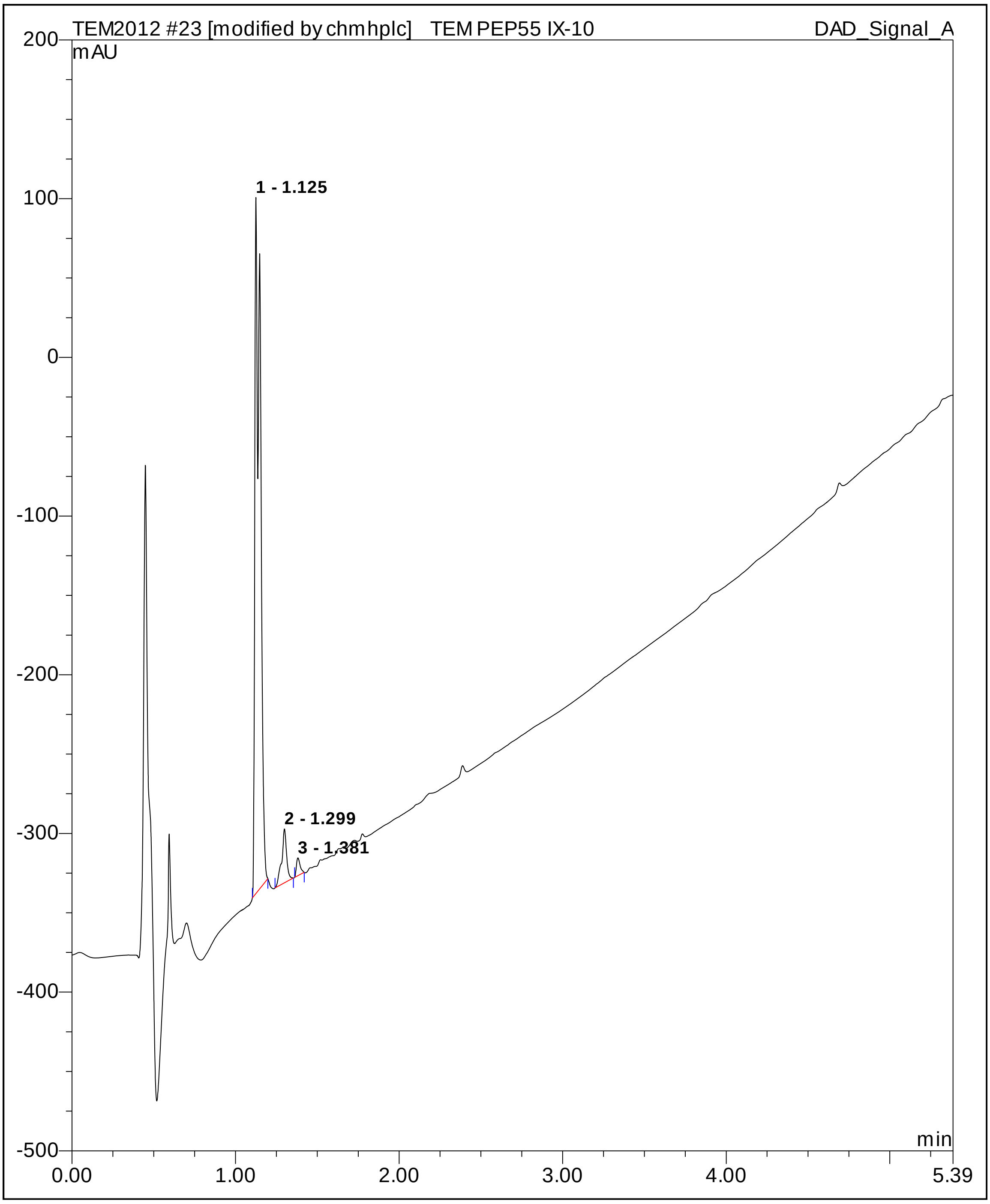

FITC-Gaba-Ser-pTyr-Val-Asn-Val-Gln-NH_2 17

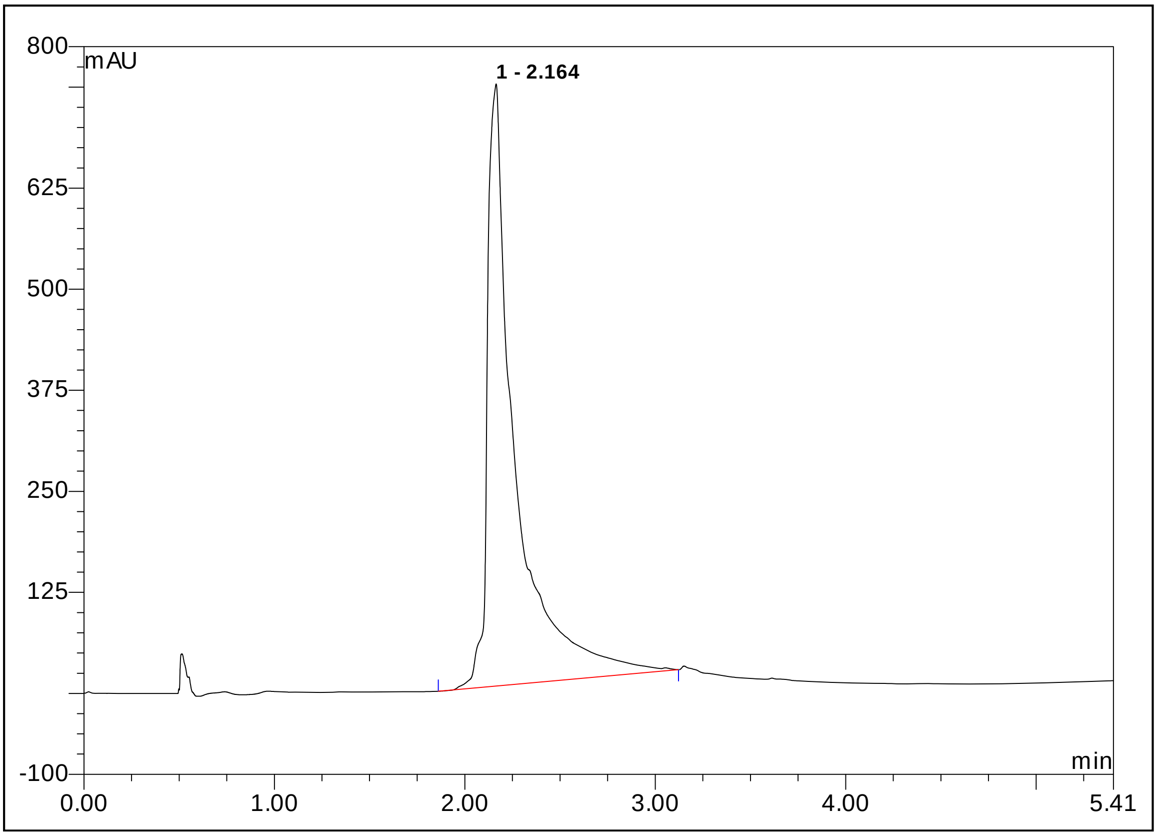

AcHN-Ser-Tyr-Val-Asn-Val-Gln-NH_2 18

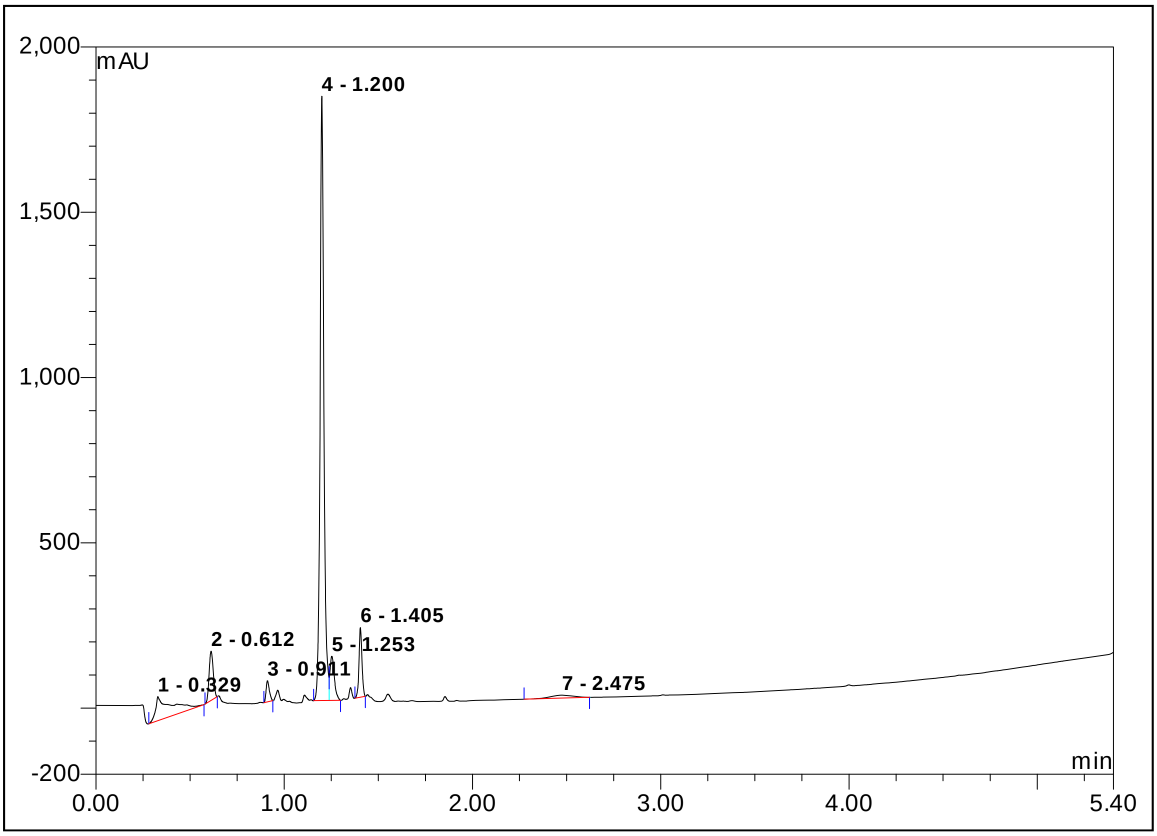

AcHN-Ser-His-Val-Asn-Val-Gln-NH_2 19

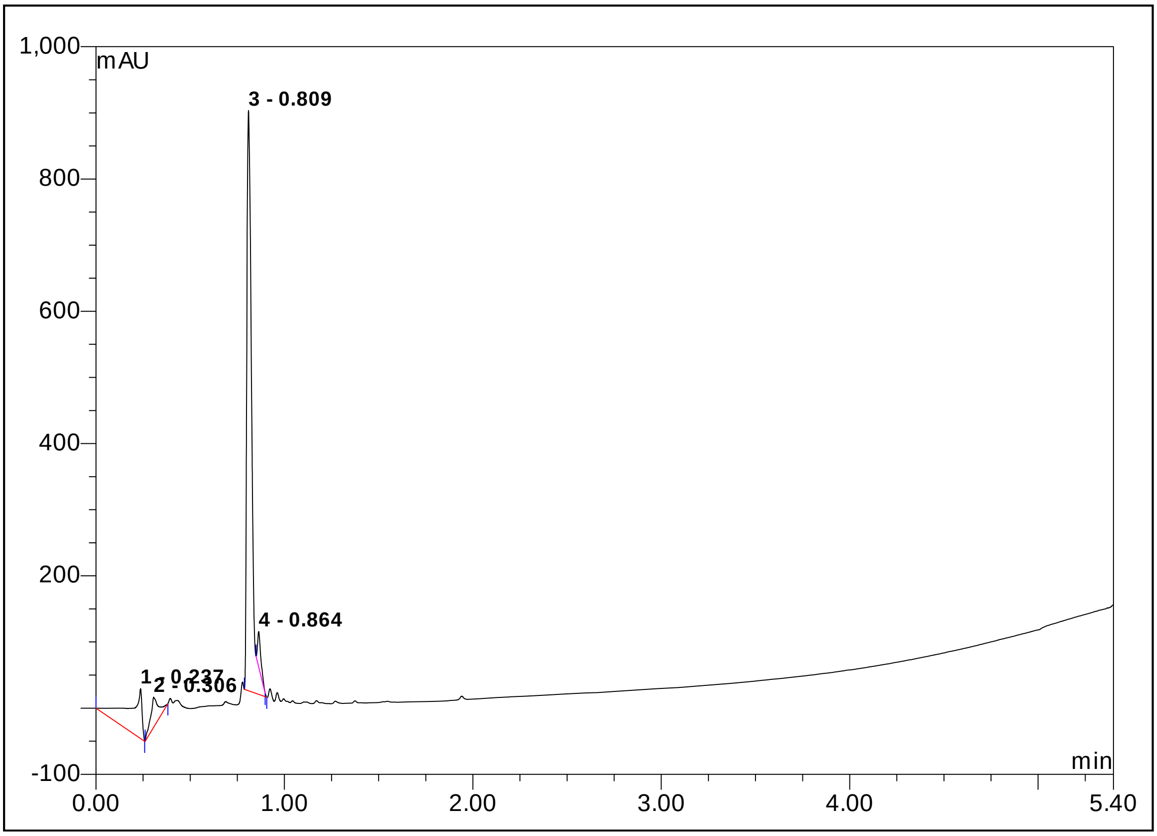

AcHN-Ser-pSer-Val-Asn-Val-Gln-NH_2 20

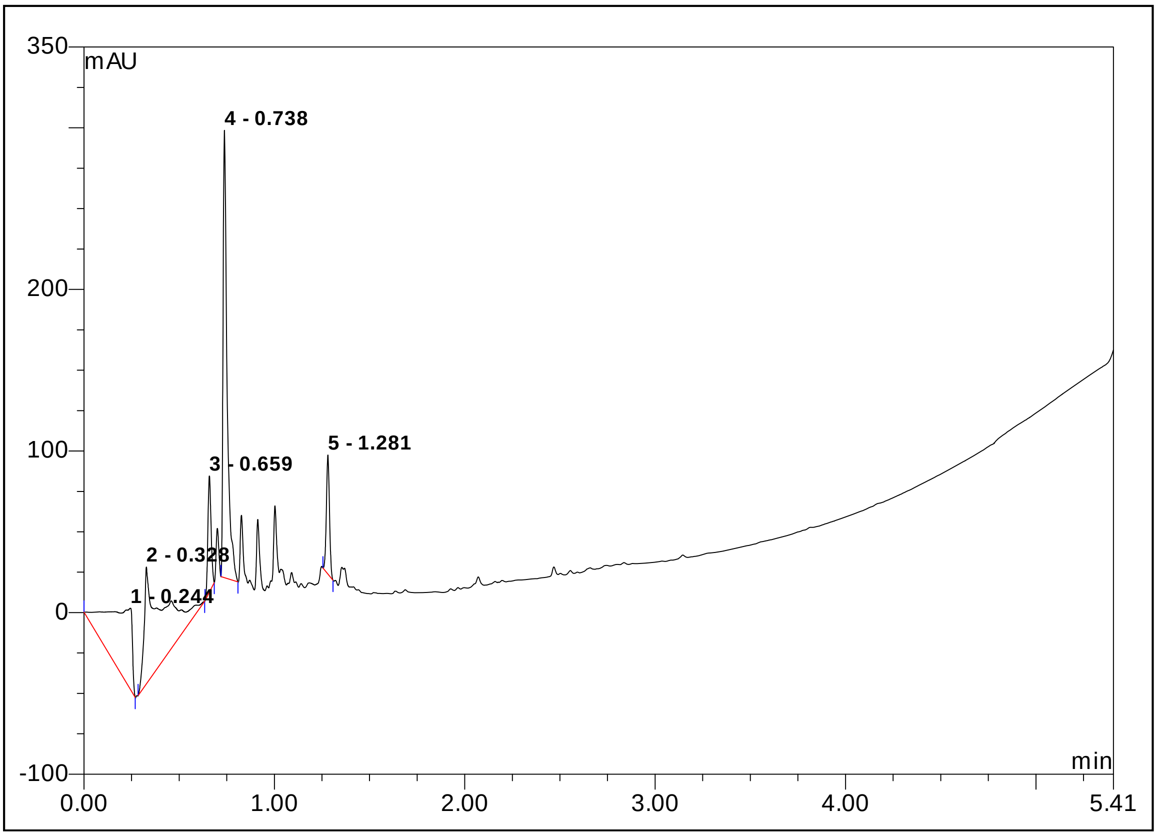

AcHN-Ser-pThr-Val-Asn-Val-Gln-NH_2 21

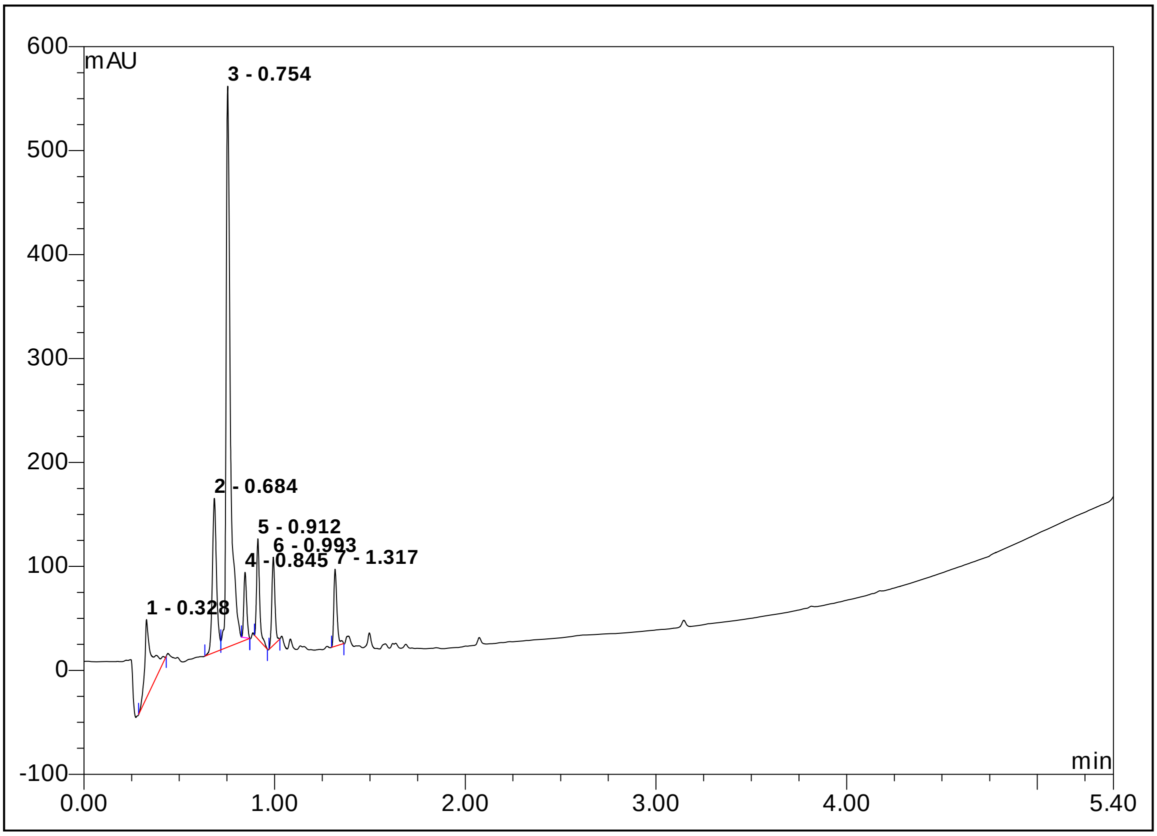

NMR Spectra

^{1}H, ^{13}C and ^{31}P NMR Spectra of **14**
^{1}H NMR spectra of **16** and **S1**

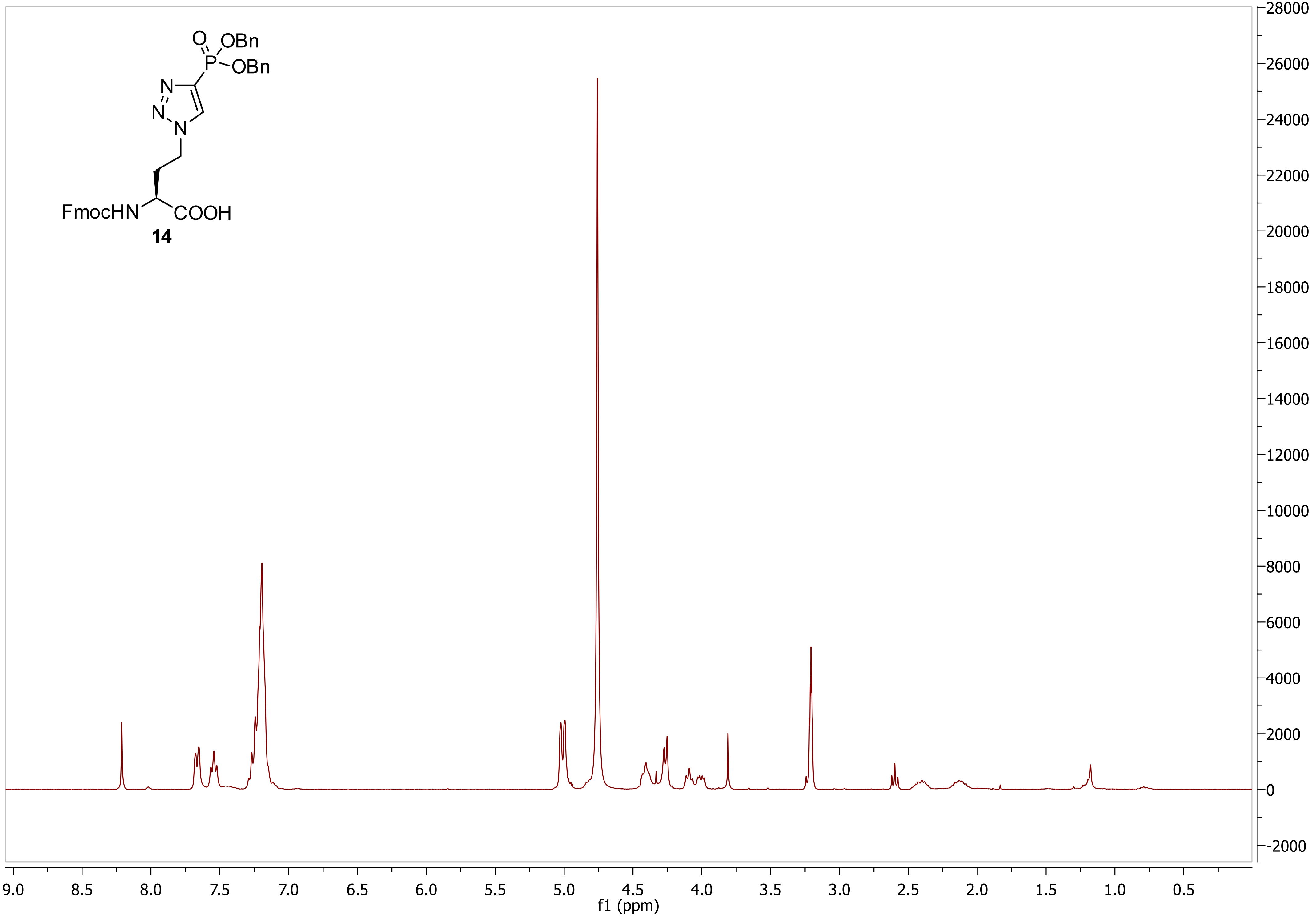

O
OBn
P
OBn
N
N
N
FmocHN
COOH
14
28000
26000
24000
22000
20000
18000
16000
14000
12000
10000
8000
6000
4000
2000
0
-2000
9.0
8.5
8.0
7.5
7.0
6.5
6.0
5.5
5.0
4.5
4.0
3.5
3.0
2.5
2.0
1.5
1.0
0.5
f1 (ppm)

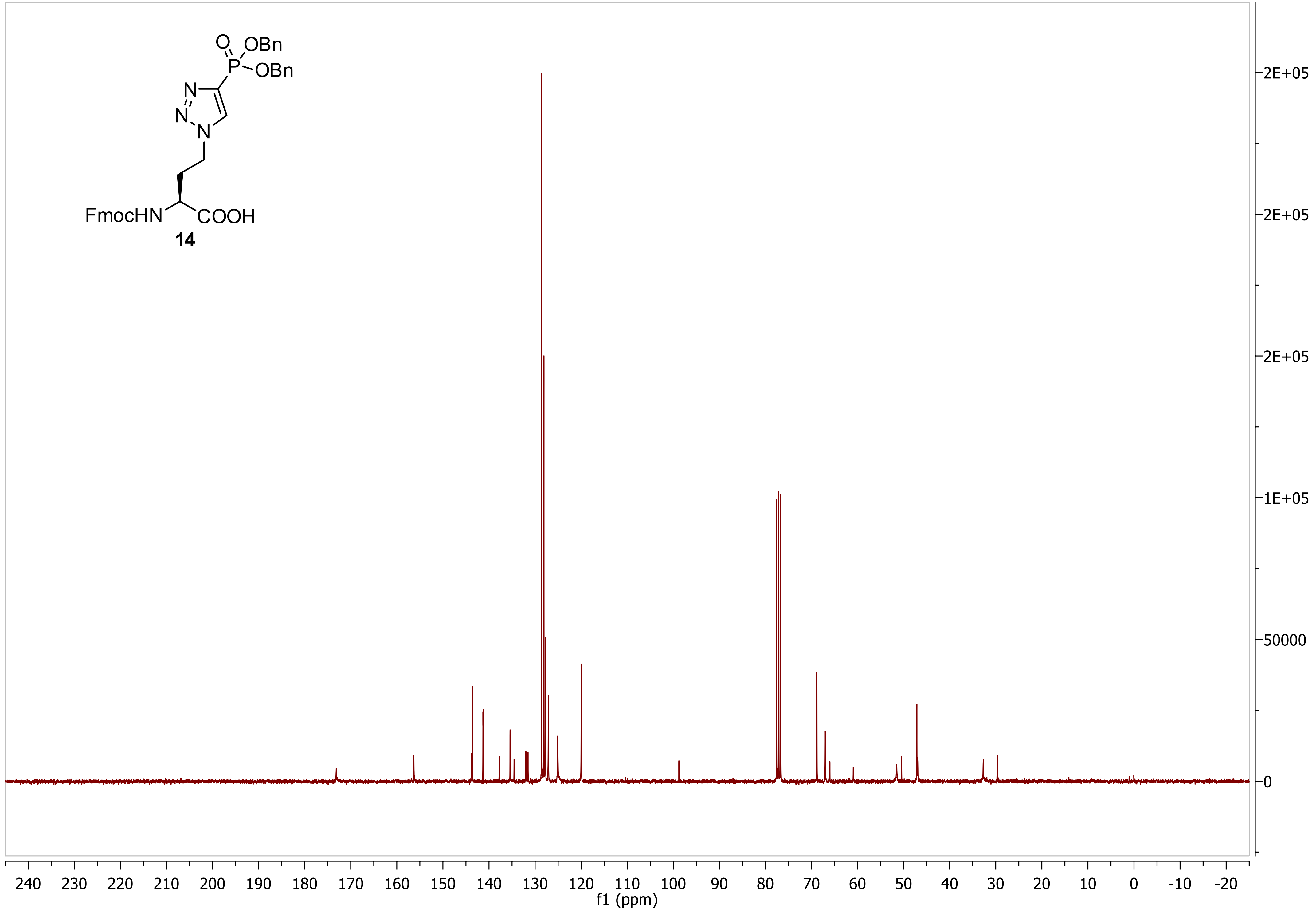
O
OBn
P
OBn
N
N
N
FmocHN
COOH
14
2E+05
2E+05
2E+05
1E+05
50000
0
240 230 220 210 200 190 180 170 160 150 140 130 120 110 100 90 80 70 60 50 40 30 20 10 0 -10 -20
f1 (ppm)

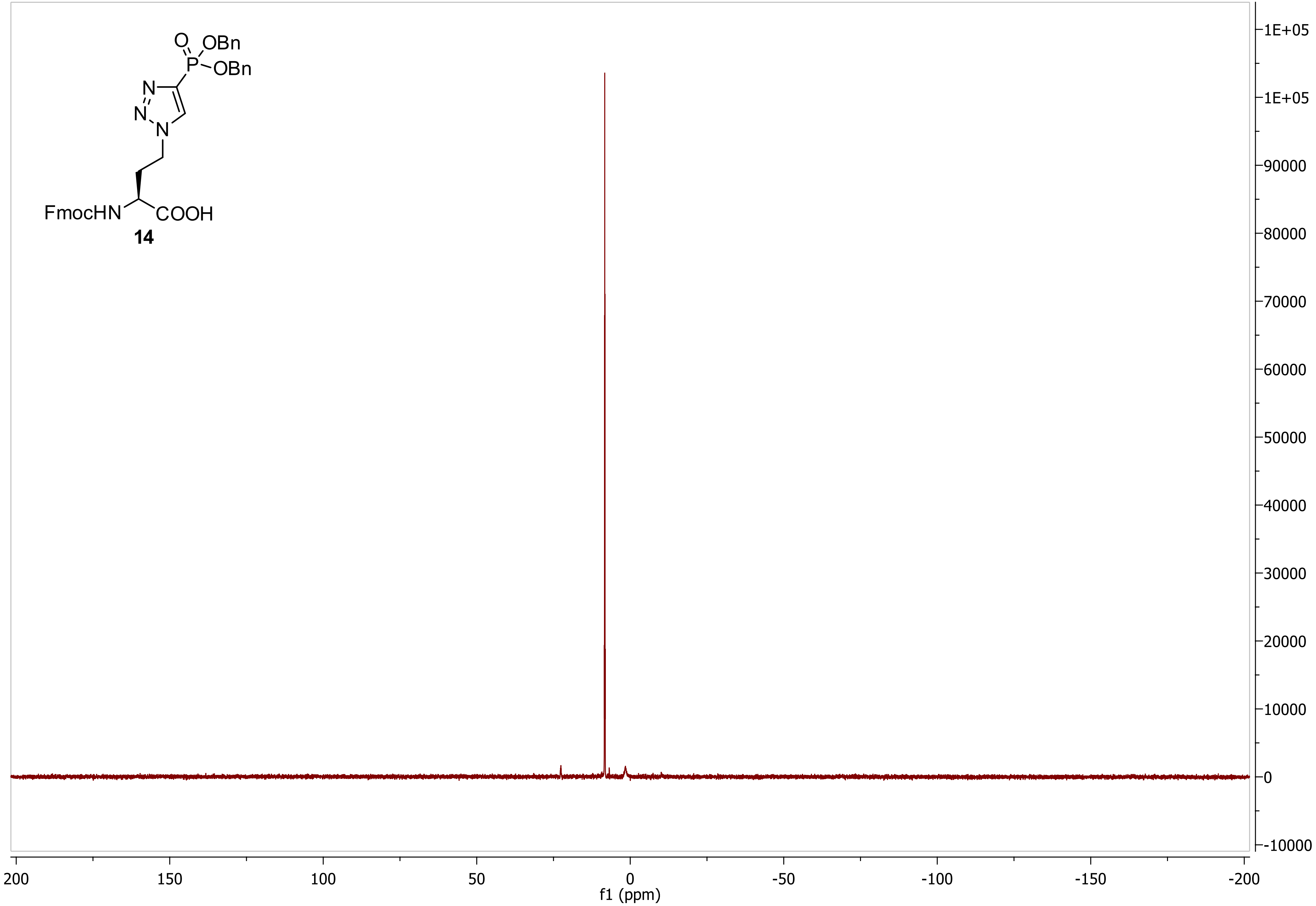
O
OBn
P
OBn
N
N
N
FmocHN
COOH
14
1E+05
1E+05
90000
80000
70000
60000
50000
40000
30000
20000
10000
0
-10000
200
150
100
50
0
-50
-100
-150
-200
f1 (ppm)

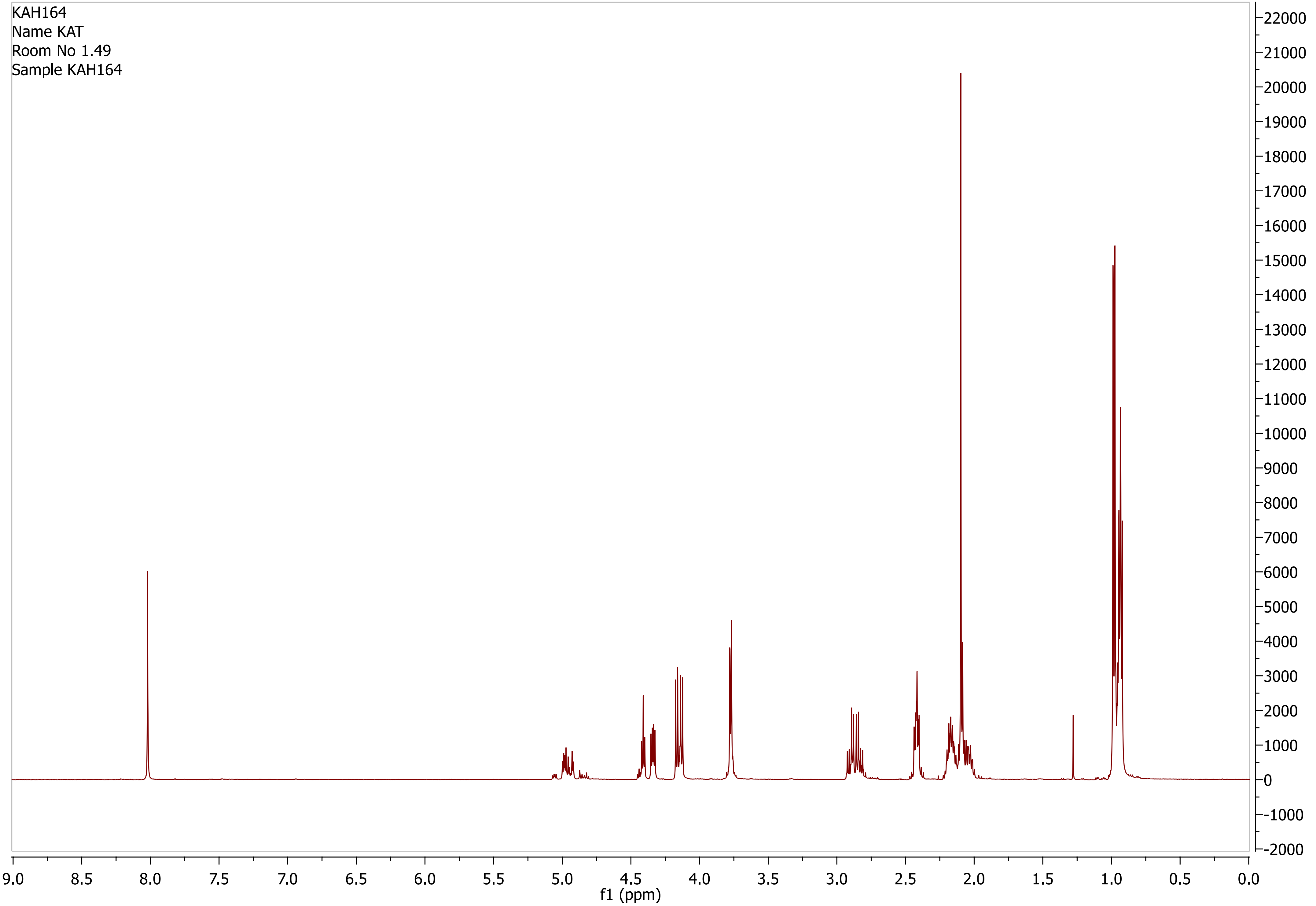
KAH164
Name KAT
Room No 1.49
Sample KAH164
f1 (ppm)

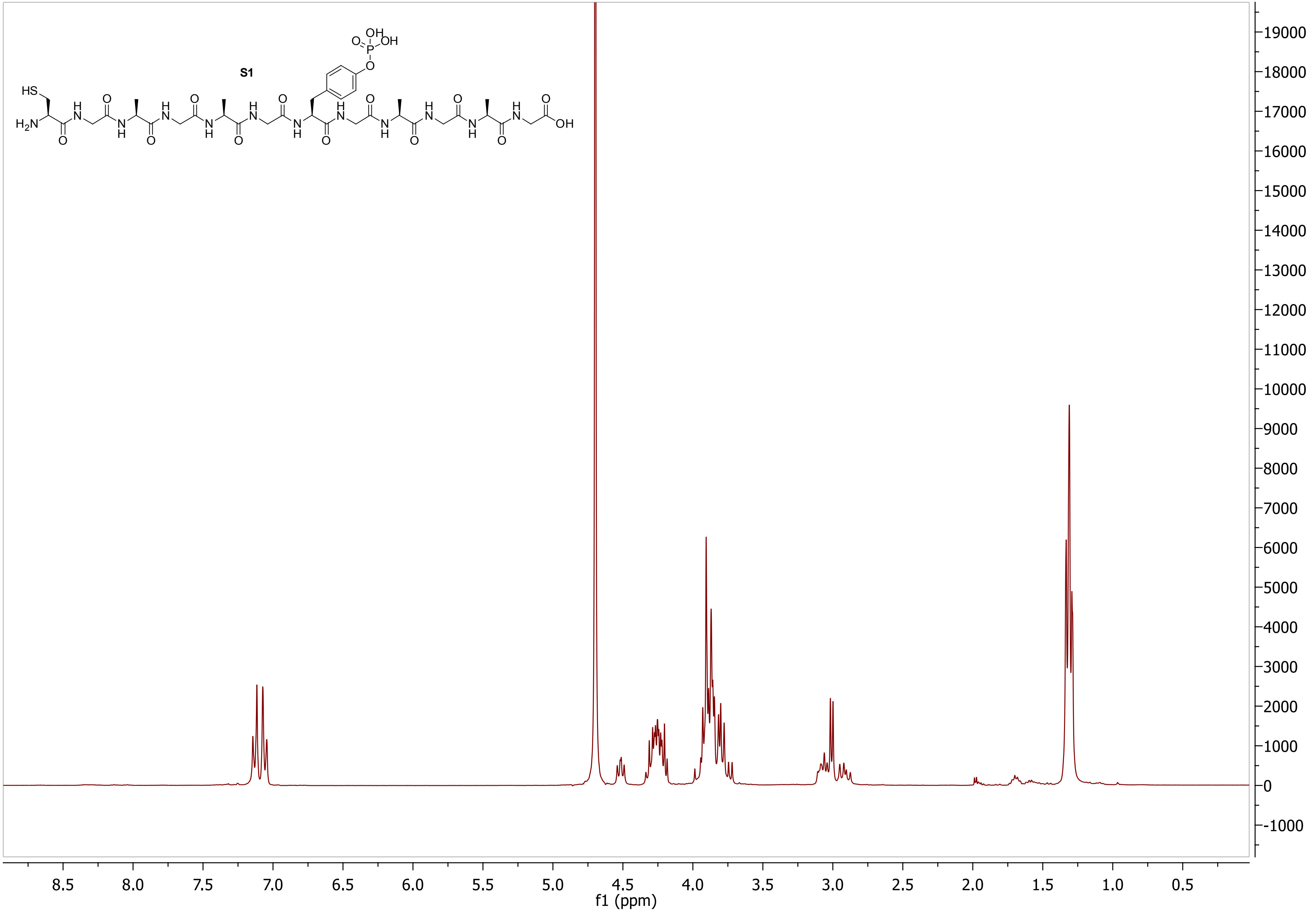
S1
f1 (ppm)
